# Human UFSP1 is an active protease that regulates UFM1 maturation and UFMylation

**DOI:** 10.1016/j.celrep.2022.111168

**Published:** 2022-08-03

**Authors:** David Millrine, Thomas Cummings, Stephen P. Matthews, Joshua J. Peter, Helge M. Magnussen, Sven M. Lange, Thomas Macartney, Frederic Lamoliatte, Axel Knebel, Yogesh Kulathu

**Affiliations:** 1 Medical Research Council Protein Phosphorylation & Ubiquitylation Unit (MRC-PPU), School of Life Sciences, University of Dundee, Dow Street, Dundee DD1 5EH, UK

**Keywords:** UFM1, ubiquitin, cysteine protease, endoplasmic reticulum, ER, ribosome, ubiquitin-like modifier, membrane protein, UFC1, UBA5

## Abstract

An essential first step in the post-translational modification of proteins with UFM1, UFMylation, is the proteolytic cleavage of pro-UFM1 to expose a C-terminal glycine. Of the two UFM1-specific proteases (UFSPs) identified in humans, only UFSP2 is reported to be active, since the annotated sequence of UFSP1 lacks critical catalytic residues. Nonetheless, efficient UFM1 maturation occurs in cells lacking UFSP2, suggesting the presence of another active protease. We herein identify UFSP1 translated from a non-canonical start site to be this protease. Cells lacking both UFSPs show complete loss of UFMylation resulting from an absence of mature UFM1. While UFSP2, but not UFSP1, removes UFM1 from the ribosomal subunit RPL26, UFSP1 acts earlier in the pathway to mature UFM1 and cleave a potential autoinhibitory modification on UFC1, thereby controlling activation of UFMylation. In summary, our studies reveal important distinctions in substrate specificity and localization-dependent functions for the two proteases in regulating UFMylation.

## Highlights

• Active human UFSP1 is translated from a non-canonical start site
• UFSP1 mediates UFM1 maturation and cleaves UFM1 off UFMylated UFC1
• Subcellular localization dictates unique substrate preferences of UFSP1 and UFSP2

## Graphical abstract

**Figure:**
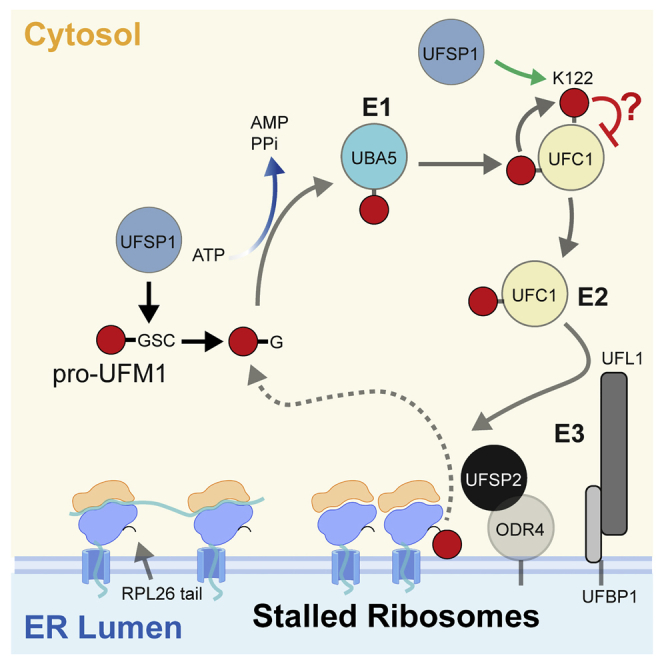

## Teaser

UFM1 requires proteolytic maturation in order to be conjugated to ribosomes; however, the enzyme responsible is unclear. Millrine et al. identify UFSP1 as the UFM1-activating peptidase and identify functions of UFSP1 in a regulatory circuit involving the E2 enzyme, UFC1.

## Introduction

The ubiquitin-like protein ubiquitin fold modifier 1 (UFM1) is emerging as a central regulator of protein homeostasis through its role in ribosome quality control (Gerakis et al., 2019; Banerjee et al., 2020; Witting and Mulder, 2021). The physiological importance of protein UFMylation is evidenced by mutations in UFM1 pathway components that result in neurodevelopmental pathophysiology including cerebellar ataxia, encephalopathy, epilepsy, peripheral neuropathy, and systemic skeletal abnormalities characterized by abnormal cartilage development (Colin et al., 2016; Duan et al., 2016; Egunsola et al., 2017; Di Rocco et al., 2018). In mice, knockout of UFM1 pathway components results in early-stage embryonic lethality that is attributed to defective hematopoiesis (Tatsumi et al., 2011). UFMylation is therefore essential for normal physiology, with evidence of involvement in tissue development. Recent studies linking UFM1 to ER stress responses and secretory pathways highlight a potential mechanism to explain these observations. Indeed, UFM1 is conjugated to ER membrane-associated ribosomal subunits (Walczak et al., 2019; Liang et al., 2020), which is induced by ribosomal stalling (Wang et al., 2020).

Produced as an 85-amino-acid precursor, UFM1 must first be proteolytically activated through the removal of a serine-cysteine dipeptide at its C terminus (Komatsu et al., 2004). Like ubiquitin, UFM1 is conjugated to substrates through an enzymatic cascade of E1 (UFM1-activating enzyme 5; UBA5), E2 (UFM1-conjugating enzyme 1; UFC1), and E3 (UFM1 specific ligase 1; UFL1) enzymes, resulting in the formation of an isopeptide bond between the C-terminal glycine of UFM1 and the substrate lysine (Komatsu et al., 2004). These core enzymes are supported by accessory factors that include UFM1-binding protein 1 (UFBP1/DDRGK1) and CDK5 regulatory subunit-associated protein 3 (CDK5RAP3), whose function is poorly defined (Yang et al., 2019; Stephani et al., 2020). UFBP1 and UFL1 localize to the ER and they are thought to catalyze the UFMylation of Ribosomal Protein L26 (RPL26) (Walczak et al., 2019; Stephani et al., 2020). UFMylation of RPL26 in proximity to the Sec61 translocon and oligosaccharyl-transferase complex occurs after ribosome stalling and initiates specialized autophagy of the ER membrane to facilitate clearance of arrested nascent peptides and ribosomes through a lysosomal pathway (Walczak et al., 2019; Liang et al., 2020; Wang et al., 2020). Termed ER-phagy, this organelle-specific degradation pathway involves the wholesale targeting of regions of the rough ER for lysosomal degradation (Khaminets et al., 2015). In a pathway dependent on mitochondrial respiration, acute amino acid starvation stimulates ER-phagy via a pathway requiring UFM1 system components and modification of RPL26 (Liang et al., 2020). The UFM1 system may, therefore, control turnover of the translational apparatus in response to the cellular and metabolic state. An elaborate system of regulation appears to have evolved solely for this purpose, as RPL26 is the most compelling UFM1 substrate described to date (Walczak et al., 2019).

While the precise function is unclear, it appears that proper functioning of the pathway requires an equilibrium of UFM1 conjugation that supports stalled ribosome clearance and ER turnover without damaging the cell’s capacity for protein biogenesis. Tight regulation is reflected in the specificity of the pathway which, unlike the highly redundant ubiquitin system, is coordinated by only a handful of enzymes (Komatsu et al., 2004; Gerakis et al., 2019; Walczak et al., 2019; Liang et al., 2020). At present only two enzymes, UFM1 specific protease 1 and 2 (UFSP1 and UFSP2), are known to cleave UFM1 conjugates, with UFSP1 reported to be catalytically inactive in humans (Kang et al., 2007; Ha et al., 2008).

UFM1 requires the peptidase-linked cleavage of the C-terminal serine^84^-cysteine^85^ peptide to achieve its mature form, a prerequisite for the attachment of UFM1 onto substrates. However, in *UFSP2*^*−/−*^ cell lines, UFM1 modification of the ribosomal subunit RPL26 is enhanced, not abolished (Muona et al., 2016; Walczak et al., 2019), leading us to challenge the notion that UFSP2 is the only UFM1-specifc protease in humans. Hence, we hypothesize that additional unreported mechanisms must exist to support UFM1 maturation in the absence of UFSP2. Indeed, the probable existence of additional UFM1-specific proteases has been noted by others (Walczak et al., 2019; Witting and Mulder, 2021). In the present study we sought to identify this unknown UFM1 peptidase. To our surprise, we isolated from cells UFSP1 that is larger than the presently annotated form and has activity toward the UFM1 precursor. Analysis of knockout cell lines identified overlapping and unique contributions of UFSP1 and UFSP2 to ribosome modification and processing of precursor UFM1. Furthermore, we identify a role for localization of UFSP2 at the ER via its interaction with the ER resident protein ODR4 for its ability to remove UFM1 from RPL26. In addition, UFSP1 is unable to reverse RPL26 UFMylation, highlighting the specificity in the system. Intriguingly, we observe a striking accumulation of UFMylated UFC1 in cells lacking UFSP1. Based on our observations, we propose dual roles for UFSP1 in activating UFMylation, first at the level of UFM1 maturation and second, by removing a potential autoinhibitory modification on UFC1. Thus, UFSP enzymes act at disparate points of the pathway to ensure appropriate UFMylation.

## Results

### UFSP2 is not the sole UFM1-specific protease in humans

**Figure 1:**
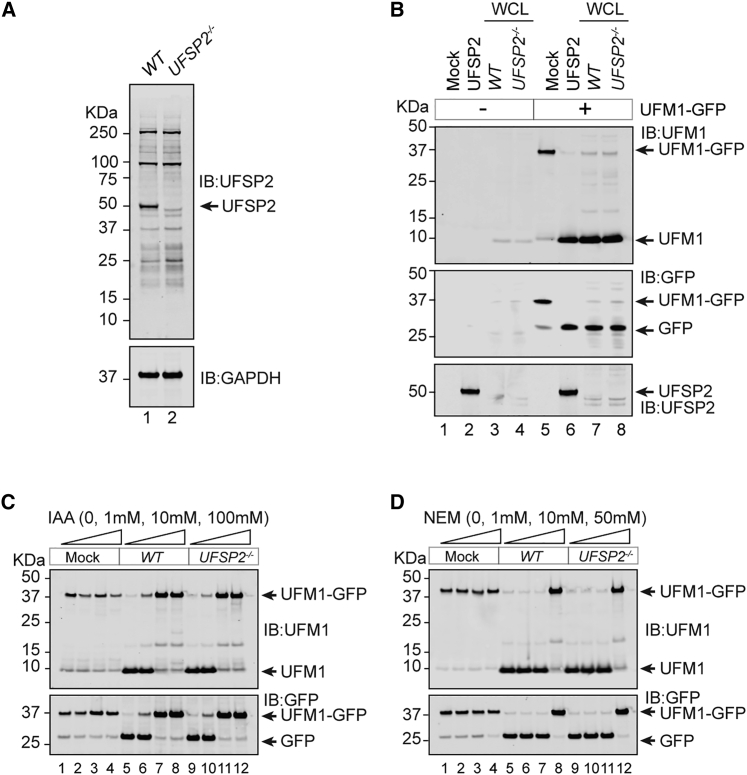
UFSP2 is not the sole UFM1-specific peptidase in human cells (A) Confirmation of UFSP2 knockout by western blotting. (B) *In vitro* assay incubating cell lysates from wild-type (WT) and *UFSP2*^−/−^ HEK293 cells with a UFM1-GFP fusion protein. Cleavage of a recombinant UFM1-GFP fusion protein into its constituent parts, UFM1 and GFP, is interpreted as peptidase activity. Recombinant UFSP2 (2 μM) is included as a positive control. (C and D) Prevention of UFM1-GFP cleavage by the cysteine peptidase inhibitors iodoacetamide (IAA, C) and *N*-ethylmaleimide (NEM, D). Cell lysates were pretreated for 1 h in darkness at room temperature prior to mixing with the recombinant UFM1-GFP probe. Probe-lysate incubations were performed at 37°C for 2 h. Data are representative of more than three independent experiments.

To confirm the existence of additional peptidases with activity toward precursor UFM1, we generated *UFSP2*^*−/−*^ cell lines using CRISPR-Cas9 approaches. Consistent with previous studies, we observed an increase in both RPL26-(UFM1)_1_ and RPL26-(UFM1)_2_ species as a result of UFSP2 deficiency (Figures 1A and S1A–S1D) (Ishimura et al., 2017; Walczak et al., 2019; Liang et al., 2020; Wang et al., 2020; Kulsuptrakul et al., 2021). We next developed an experimental system to monitor UFM1 peptidase activity, whereby cell lysates were incubated with a reporter protein comprising pro-UFM1^1−85^ fused at its carboxy terminus to GFP via a short peptide linker. Intriguingly, lysates derived from parental wild-type (WT) and *UFSP2*^*−/−*^ HEK293 cells showed equivalent ability to cleave the UFM1-GFP fusion construct to liberate mature UFM1, suggesting the presence of an additional protease (Figures 1B and S1E). Cleavage of the fusion protein could be prevented by preincubation with broad-acting cysteine peptidase inhibitors iodoacetamide or *N*-ethylmaleimide, suggesting that the proteolytic activity observed in *UFSP2*^*−/−*^ cells is due to a cysteine-based protease (Figures 1C and S1E). Taken together, these data reveal the existence of additional UFM1-targeting cysteine peptidases with the capacity to activate precursor UFM1.

### Isolation of additional UFM1 peptidase activity from human cells

**Figure 2:**
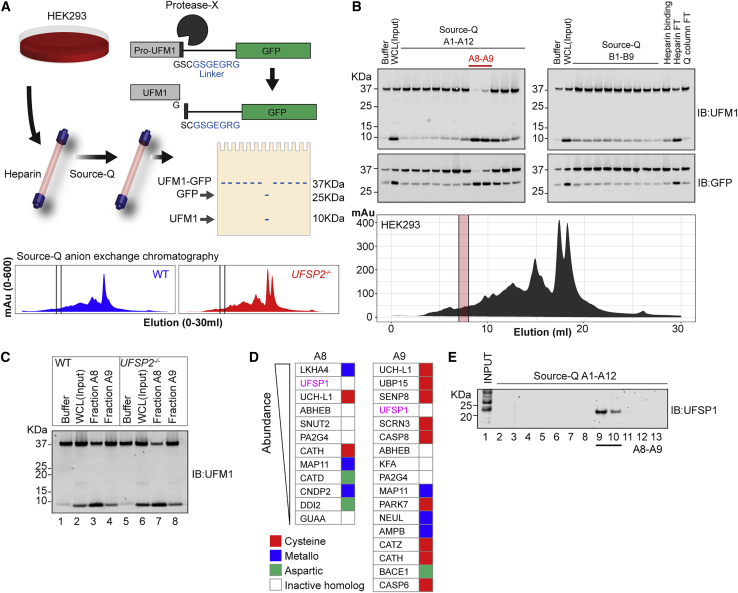
Screening for alternative UFM1 protease identifies UFSP1 as a candidate peptidase (A) (Top) Schematic overview of the screening process. HEK293 cells are lysed by mechanical stress and fractionated sequentially over heparin and Source-Q columns. Eluted fractions are screened for activity by incubation with the UFM1-GFP fusion protein. (Bottom) Chromatograms showing protein eluted from Source-Q columns on a salt gradient. Shown are experiments performed in parallel using unmodified HEK293 wild-type (WT) (blue) and *UFSP2*^*−/−*^ (red) HEK293 cells. (B) Representative screening results. Heparin-binding proteins have been eluted in a single fraction (“Heparin binding”) while Source-Q-binding proteins have been eluted in fractions A1–B9. Eluted fractions were incubated with UFM1-GFP fusion protein for 2 h at 37°C and analyzed by immunoblot (IB). Heparin and Q-column flow-through (FT) are shown on the far right. Protein cleavage activity is detected in Q-column fractions A8 and A9 (red lines). (C) *In vitro* assay incubating active fractions (A8 and A9) purified from WT and *UFSP2*^*−/−*^ HEK293 cell lysates with the UFM1-GFP fusion protein. Experiment is the same as depicted by chromatograms in (A). (D) Identity of proteases identified in the active fractions. The output of mass spectrometry analysis of the active fractions was aligned with MEROPS database annotations to identify proteases. (E) Immunoblot analysis of endogenous UFSP1 in active fractions.

We next employed biochemical approaches to identify the enzyme responsible. Lysates from HEK293 cells were fractionated sequentially over heparin and Source-Q columns, and ensuing fractions were screened for peptidase activity by monitoring cleavage of the UFM1-GFP reporter (Figure 2A). UFM1-specific peptidase activity was restricted to two sequential fractions and, strikingly, these fractions did not have detectable amounts of UFSP2 (Figures 2B and S2A). Importantly, this activity could be recapitulated in fractionations using *UFSP2*^*−/−*^ cells (Figure 2C). We were therefore successful in enriching additional UFM1 peptidase activity, distinct from UFSP2.

To identify the protease, we adopted an unbiased approach using liquid chromatography-mass spectrometry (LC-MS) profiling of the active fraction from *UFSP2*^*−/−*^ cells. These data identified 974 proteins, including 21 proteins documented as having deubiquitinase or hydrolase activity (Figure S2B). Among these, ubiquitin carboxy-terminal hydrolase L1 (UCHL1) distributed in close alignment with the novel peptidase activity (Figure S2C), an attractive candidate given its historic association with the maturation of ubiquitin precursors (Grou et al., 2015). However, when tested, neither recombinant UCHL1 nor related family members (UCHL3, UCHL5, or BAP1) could cleave the UFM1-GFP reporter (Figure S2D). Hence, we depleted UCHL1 using CRISPR-Cas9 and repeated the fractionation and MS analyses (Figures S2E–S2G). To our surprise, these analyses revealed UFM1-specific peptidase-1 (UFSP1), characterized as an inactive homolog of UFSP2, among the top candidates in both active fractions (Figure 2D). Analysis by immunoblotting confirmed the restricted distribution of UFSP1 in the two active fractions (Figure 2E). Therefore, our cell-fractionation studies successfully captured an active form of UFSP1, a surprising observation considering the present consensus surrounding UFSP1 non-functionality in humans (HUGO Gene Nomenclature Committee [HGNC] annotation).

### Human UFSP1 is an active cysteine protease

**Figure 3:**
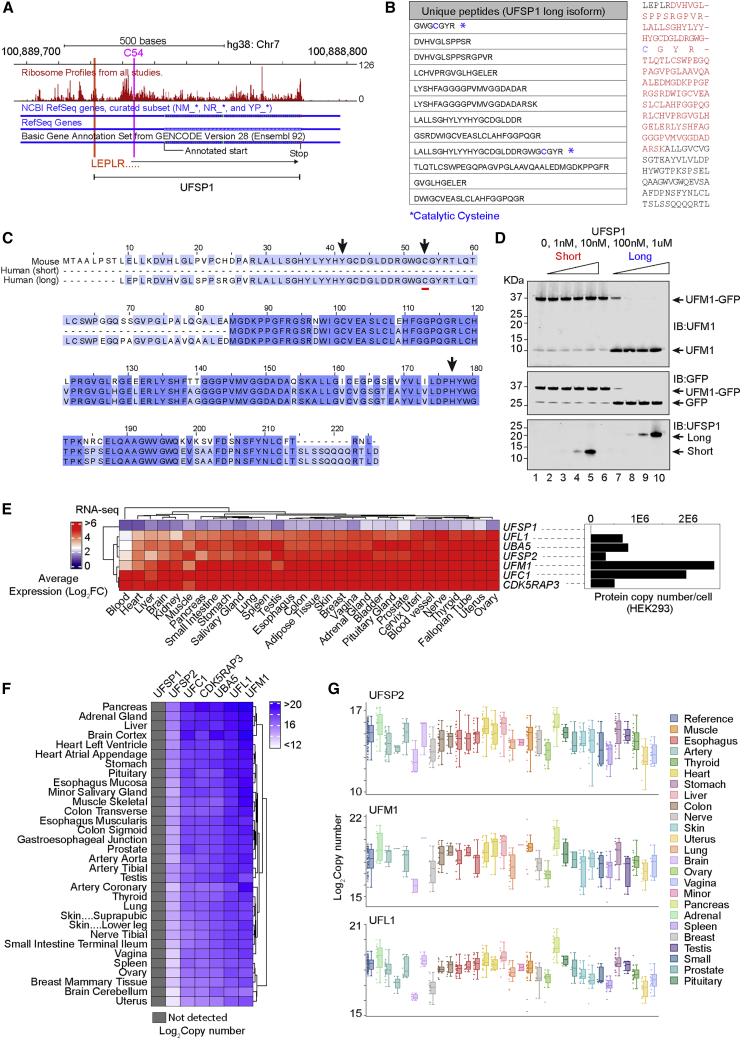
UFSP1 translated from non-canonical start site is an active protease (A) Ribosome profiling data downloaded from GWIPS-viz (https://gwips.ucc.ir/). Global aggregate protected reads from multiple studies are shown in red (UCSC-track) aligned to Refseq and GENCODE (v28) gene annotation. (B) (Left) LC-MS data showing peptides mapping to long-isoform UFSP1 identified in active fractions. (Right) Amino acid sequence of proposed full-length UFSP1. Sequences in red are identified in LC-MS analysis of active fractions. The catalytic cysteine is highlighted blue. (C) Cross-species multiple sequence alignment of UFSP isoforms. Human short (Q6NVU6), human long (A0A5F9ZGY7), and mouse (Q9CZP0) UFSP1. (D) *In vitro* assay incubating recombinant long- and short-isoform UFSP1 variants with the UFM1-GFP fusion protein. (E) (Left) RNA-sequencing analysis of human tissues by the Genotype Tissue (GTEx) consortium (https://gtexportal.org/home/). Shown are the log_2_ transformed transcripts per million. (Right) Data independent acquisition quantitative proteomics analysis of UFM1 pathway components in HEK293 cells. (F and G) Copy number estimation of UFM1 pathway components in human tissues derived from published proteomics data (PXD016999). Heatmaps are clustered using the Euclidean method.

The UFSP1 activity we observe raises the possibility that the HGNC annotation is incorrect and the UFSP1 expressed in cells spans a longer stretch at the N terminus that contains the catalytic residues. Aggregate ribosome profiling data across multiple studies (Ribo-seq; GWIPS-viz; https://gwips.ucc.ie/) supported our hypothesis that regions upstream of the annotated *UFSP1* locus are actively translated. A high number of protected reads was observed 5′ to the incorrect start site with coverage of the catalytic cysteine (C54) and a putative CTG start codon (Figure 3A). Reported previously, this non-canonical initiation site is a rare example of translation initiation from codons other than ATG and is proposed to be the only in-frame start codon capable of producing a functional UFSP1 cysteine protease (Ivanov et al., 2011). We therefore reanalyzed MS data from the two active fractions to search for peptides that correspond to regions upstream of the annotated start site. This analysis identified 12 matching peptides corresponding to the N terminus of UFSP1 (65% coverage) and included the catalytic cysteine (Figure 3B). Indeed, close inspection of curated isoforms of UFSP1 (Uniprot, Ensembl, and NCBI) showed that of the two human UFSP1 variants, one isoform (A0A5F9ZGY7) shares amino acid residues described as essential for the catalytic activity of murine UFSP2 (Figure 3C) (Kang et al., 2007; Ha et al., 2008). This conclusion is supported by cross-species bioinformatic analysis of *UFSP1* transcripts that support the existence of the longer version (Figures S3A–S3C). Further confirmation is obtained in immunoblotting of endogenously expressed UFSP1, which migrates at a size consistent with the predicted molecular mass (∼24 kDa) upon translation of the correct transcript (Figure 2E). Importantly, this is larger than the incorrectly HGNC-annotated UFSP1 (15 kDa).

These results imply that the long isoform is the primary UFSP1 protein expressed in human cells that is catalytically active. To test this experimentally, we expressed and purified recombinant UFSP1 corresponding to the 24 kDa form and the incorrectly annotated protein (Figure S3D). When incubated with the UFM1-GFP fusion protein, only the recombinant long-isoform UFSP1, but not its truncated form, showed cleavage activity *in vitro* (Figure 3D). Of note, UFSP1 is expressed at very low levels in cells as judged by RNA-sequencing data (GTEx) and quantitative proteomics data (Figures 3E–3G), possibly explaining why the correct UFSP1 may have escaped detection, further contributing to the acceptance of the misannotated form. Taken together, our analyses provide convincing evidence that the long-isoform UFSP1 we here identify is the correct endogenous UFSP1 that is catalytically competent via a cysteine-thiol-based mechanism, thus correcting the long-held misconception that human UFSP1 is an inactive protease.

### Characterization of UFSP1

**Figure 4:**
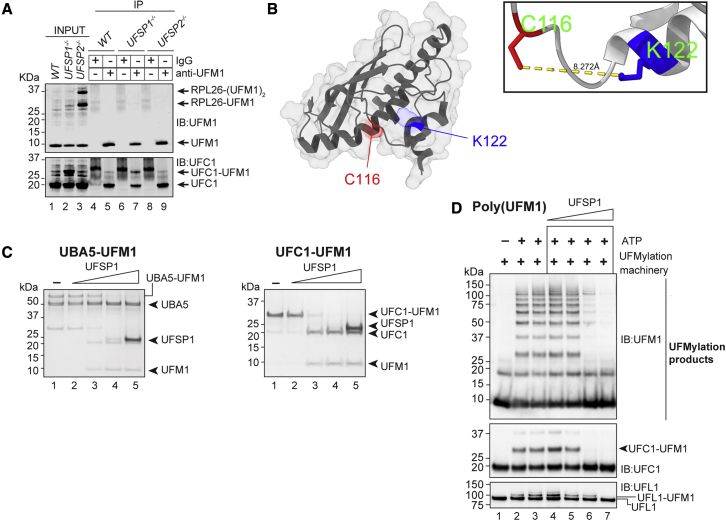
UFSP1 is active against diverse substrates *in vitro* (A) Immunoprecipitation (IP) of UFM1 from the indicated cell lysates. (B) Crystal structure of UFC1 (PDB: 2Z60) with K122 (blue) and C116 (red) highlighted. (C) Activity of recombinant UFSP1 against the indicated substrates. (D) Activity of UFSP1 against UFMylation products. UFM1 pathway components were reconstituted *in vitro* (UBA5, UFC1, UFBP1-UFL1) in the presence of ATP. After 1 h the reaction was quenched with apyrase and incubated with increasing molar concentrations of recombinant UFSP1.

Since nothing is known about the function of UFSP1 in cells, we next sought to identify the roles of UFSP1 in regulating UFMylation and to dissect the relative contributions of UFSP1 and UFSP2. First, a comparison of UFMylation in *UFSP1*^*−/−*^ and *UFSP2*^*−/−*^ cells revealed an increase of RPL26 UFMylation in UFSP2^*−*^^*/*^^*−*^ cells, which was missing in the WT and *UFSP1*^*−/−*^ cells. Instead, *UFSP1*^*−/−*^ cells showed an increase in UFMylated UFC1 that could be confirmed in immunoprecipitations of UFM1 (Figure 4A). To confirm these observations and identify the lysine residue on UFC1 that is modified, we immunoprecipitated UFM1 from *UFSP1*^*−/−*^ cell lines and analyzed them by LC-MS. Importantly the immunoblotting data were corroborated by detection of MS spectra corresponding to UFC1 peptides in which Lys122 was modified by Val-Gly (the C-terminal UFM1 dipeptide that remains on UFMylated residues after tryptic digest) (Figure S4A). Hence, in the absence of UFSP1, there is an accumulation of UFC1 UFMylated at K122. Interestingly, K122 is situated near the catalytic cysteine of UFC1 (Figure 4B). Immunoblot analysis of cell lysates revealed a basal level of UFMylated UFC1 across all cell lines tested (n = 15) (Figure S4B). While this observation was true of both human and murine cell lines, we note the presence of an unannotated short-isoform variant of UFC1 unique to mice (Figures S4B and S4C).

While the earlier assays used a UFM1-GFP reporter to reveal peptidase activity in UFSP1, to confirm that UFSP1 has isopeptidase activity, we performed a series of *in vitro* assays with various *in vitro* generated substrates including UBA5 and UFC1 modified with UFM1 via an isopeptide bond. Furthermore, through the reconstitution of UFM1 pathway components, we were able to synthesize UFMylated products (Peter et al., 2022). These are a heterogeneous mixture of UFM1 conjugates including K69-linked polyUFM1 chains in addition to automodified UFC1 and UFL1(Peter et al., 2022). UFSP1 effectively cleaved UFM1 from these different substrates and catalyzed the disassembly of polyUFM1 chains *in vitro* (Figures 4C and 4D). Taken together, these data show that in addition to its peptidase function in targeting precursor UFM1, UFSP1 is an effective isopeptidase with activity toward diverse substrates.

### UFSP1 and UFSP2 act at separate points in the UFM1 pathway

**Figure 5:**
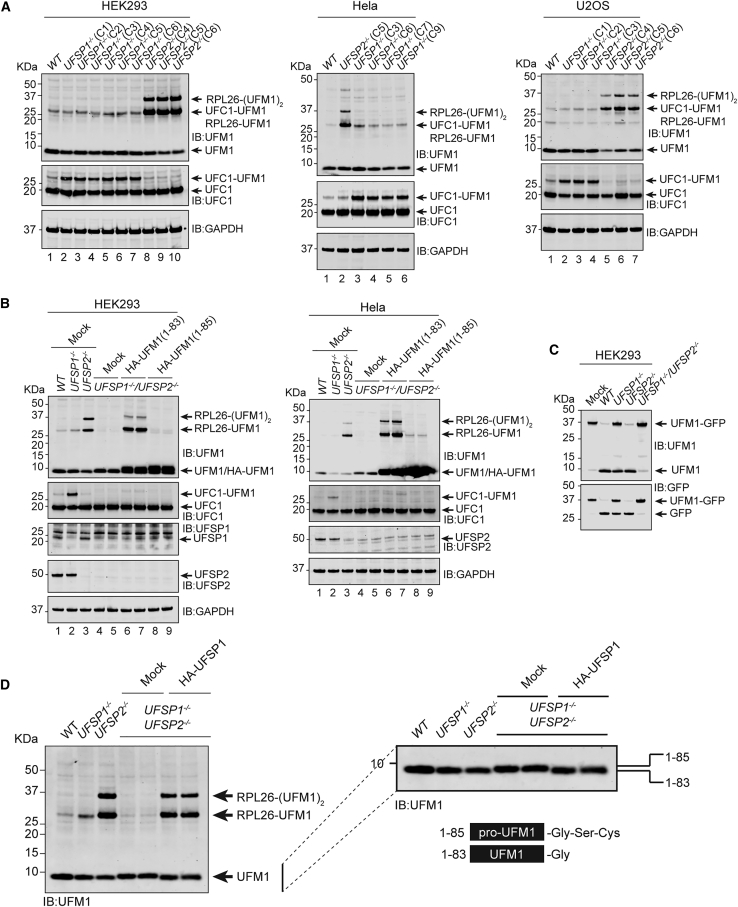
UFSP1 is the UFM1-activating peptidase *in vivo* (A) Immunoblot analysis of *UFSP1*^*−/−*^ and *UFSP2*^*−/−*^ cell lines as indicated. Labels include abbreviated clone IDs (e.g., C1 is clone 1). (B) Rescue of RPL26 UFMylation by expression of mature UFM1. Constructs expressing HA-tagged mature (UFM1^1−83^) or precursor (UFM1^1−85^) UFM1 were transiently transfected into UFSP1^−/−^/UFSP2^−/−^ double knockout cell lines. Twenty-four hours later cells were lysed and analyzed by immunoblot with the indicated antibodies. (C) *In vitro* assay incubating HEK293 cell lysates from the indicated knockout cell lines with the UFM1-GFP probe. (D) Immunoblot analysis of HEK293 cells transiently transfected with HA-tagged UFSP1. (Right) zoomed-in section of the blot shown on the left to highlight changes in electrophoretic mobility of UFM1. This western blot is reproduced complete with loading controls in Figure S5B.

To further explore the observation of enriched K122-modified UFC1 and establish that these observations are not cell line specific, we generated a series of *UFSP1* and *UFSP2* knockouts in three different cell lines, HEK293, U2OS, and HeLa, and multiple clones were confirmed by sequencing (Figure S5A). Consistent with our earlier observation (Figures S1C and S1D), we observe increased mono- and di-UFMylated RPL26 in *UFSP2*^*−/−*^ but not *UFSP1*^*−/−*^ cell lines (Figure 5A). Meanwhile, in all the different *UFSP1*^*−/−*^ cell lines tested, we observed a size shift in UFC1 of approximately 10 kDa (Figure 5A). This size shift corresponded to modification of UFC1 with UFM1 and accounted for up to 50% of cellular UFC1 in *UFSP1*^*−/−*^ cell lines (Figure 5A). These results suggest that UFC1 is constitutively modified with UFM1 in cells whose removal depends on UFSP1. By contrast, we observed no effect of loss of UFSP1 or UFSP2 on UFMylation of reported UFM1 substrates P53 and histone H4 (Figures S5B and S5C) (Qin et al., 2019; Liu et al., 2020).

In the absence of either UFSP1 or UFSP2, cells appear able to generate sufficient mature UFM1. However, in cell lines lacking both UFSP enzymes (*UFSP1*^*−/−*^/*UFSP2*^*−/−*^), a complete loss-of-function phenotype is manifested by a total absence of detectable UFMylation (Figure 5B). This could be rescued through recombinant overexpression of UFSP1 (Figure S5D). These data are consistent with a requirement for either UFSP1 and/or UFSP2 in the generation of mature UFM1 and suggest that both enzymes contribute, in a partially redundant manner, to precursor UFM1 maturation. To confirm that the complete loss of UFMylation observed in double knockout cell lines stems from an absence of mature UFM1 in these cells, *UFSP1*^*−/−*^/*UFSP2*^*−/−*^ cell lines in HEK293 and HeLa backgrounds were transiently transfected with constructs expressing either mature UFM1^1–83^ or its precursor counterpart (UFM1^1−85^). Overexpression of mature UFM1, but not proUFM1, successfully rescued mono- and di-UFMylated RPL26 (Figure 5B). These data are consistent with *in vitro* analysis of peptidase activity in cell lysates where cell lysates from *UFSP1*^*−/−*^, *UFSP2*^*−/−*^, and *UFSP1*^*−/−*^/*UFSP2*^*−/−*^ HEK293 cell lines were incubated with the proUFM1-GFP probe. Here, cleavage activity was only completely abolished in the absence of both enzymes (Figure 5C). Furthermore, close inspection of immunoblot analyses reveals a size shift in UFM1 electrophoretic mobility consistent with an increase in molecular weight corresponding to proUFM1 in cell lines lacking both UFSP enzymes (Figure 5D). While these experiments clearly demonstrate complete loss of UFM1 maturation in cells lacking both UFSP1 and UFSP2, this does not preclude the existence of additional proteases in specific cell types.

### Subcellular localization regulates function of UFSPs

**Figure 6:**
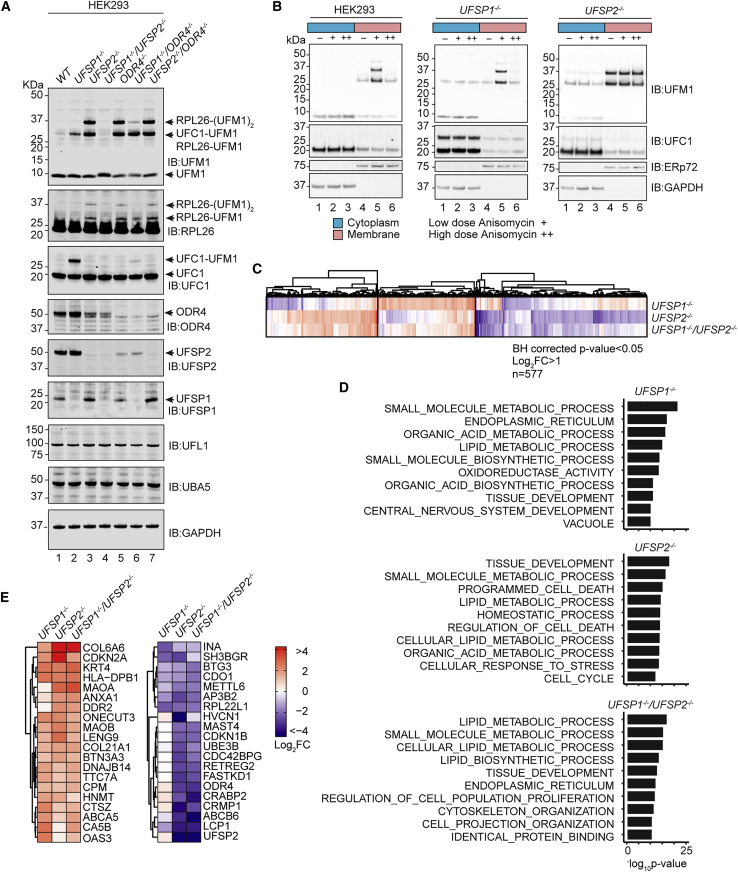
Distinct substrates and functions for the UFSPs (A) Immunoblot analysis of the indicated knockout cell lines (HEK293 Flp-in TREx). (B) Cell lines were treated with 200 nM (+) or 50 μM (++) anisomycin for 20 min prior to cell lysis. Immunoblot analysis of cytoplasmic and membrane fractions is shown. (C–E) Comparison of total proteomes of indicated cell lines. (C) Heatmap showing Log_2_ fold change (FC) in abundance of proteins passing statistical thresholds in at least one experimental condition (Benjamini-Hochberg adjusted p < 0.05; log_2_ FC > 1). Heatmap is clustered using the k-means method (n = 3). Data for all significant proteins (p_adj_ < 0.05) are shown in Figures S6D and S6E. (D) Gene ontology (GO) enrichments calculated using a hypergeometric distribution test (Broad Institute; http://www.gsea-msigdb.org/gsea/msigdb/compute_overlaps.jsp). Top ten GO enrichments are shown for each dataset. (E) Proteins subject to the most extreme changes (top/bottom ten). Proteomics data show LIMMA differential analyses (*UFSP1*^−/−^ versus WT, *UFSP2*^−/−^ versus WT, *UFSP1*/*UFSP2*^−/−^ versus WT). Volcano plots and principal component analyses are included in supplemental information. Heatmaps in (C) and (E) are shown at the same scale.

We next explored whether differences in subcellular localization of UFSP1 and UFSP2 might contribute to the differences in substrate specificities observed in knockout cells. UFSP2 interacts with odorant response abnormal protein-4 (ODR4), a transmembrane protein that is localized to the ER membrane where it is thought to anchor UFSP2 in proximity to the ER-ribosome interface (Chen et al., 2014). In contrast, sequence analysis and structural predictions suggest that UFSP1 will not interact with ODR4 and is likely to instead reside in the cytosol. This makes it more possible for UFSP1 to be the protease mainly responsible for UFM1 maturation. To disrupt the ER localization of UFSP2, we generated ODR4 knockout cell lines and monitored UFMylation (Figures 6A and S6A). Intriguingly, UFSP2 protein levels are markedly reduced in *ODR4*^*−/−*^ cell lines and vice versa, suggesting that UFSP2 and ODR4 are engaged in a mutually stabilizing relationship (Figure 6A). Moreover, UFSP2 and ODR4 knockout cell lines were an exact phenocopy in their role in restraining levels of RPL26 UFMylation (Figure 6A). In cell lines lacking both UFSP1 and ODR4, levels of di-UFMylated but not mono-UFMylated RPL26 were reduced. Interestingly, this is similar to our observation of a cell line heterozygously deficient for UFSP1 (*UFSP1*^+*/−*^/*UFSP2*^*−/−*^) where the second UFM1 modification on RPL26 was absent (Figures 6A and S6B). These data may indicate a preference for mono-UFMylated RPL26 in circumstances where mature UFM1 is limiting and aligns with the description of RPL26 modification as sequential with K132 UFMylation dependent on prior modification of K134 (Walczak et al., 2019). Finally, we investigated whether UFSP1 could contribute directly to the UFMylation pathway in ribosomal quality control. Upon induction of ribosome stalling following treatment with low-dose anisomycin, RPL26 UFMylation is induced only in the membrane fraction of WT cells (Figure 6B, top). In the absence of UFSP2, significant RPL26 UFMylation is observed, which does not increase further upon ribosome stalling (Figure 6B, bottom). In contrast, UFSP1 knockout cells remained competent for the induction of ribosome UFMylation upon ribosome stalling (Figure 6B). These data suggest that ribosome UFMylation is dynamic and is mainly regulated by UFSP2. UFSP1, on the other hand, indirectly regulates RPL26 UFMylation by facilitating UFM1 maturation and cleaving UFM1 from UFMylated UFC1.

**Figure 7:**
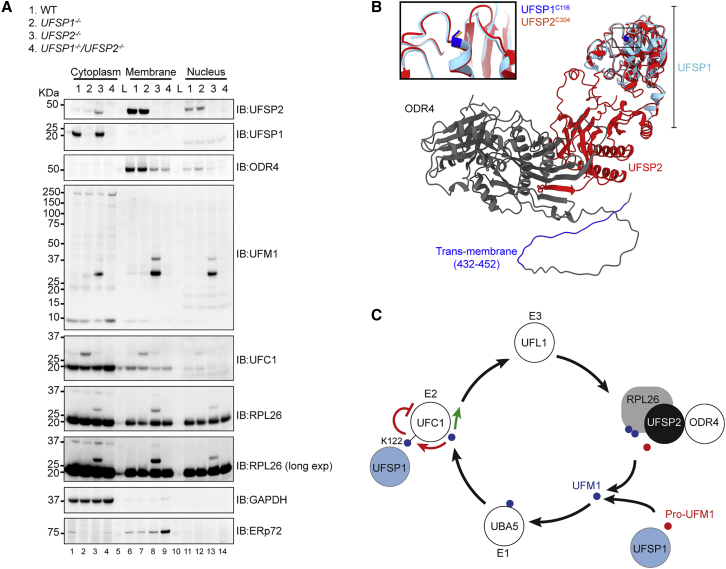
Distinct intracellular localization of UFSPs determines function (A) Immunoblot analysis of membrane, cytosol, and nuclear fractions derived from *UFSP1*^*−/−*^, *UFSP2*^*−/−*^, and *UFSP1*^*−/−*^/*UFSP2*^*−/−*^ cell lines. (B) AlphaFold prediction of UFSP2-ODR4 complex aligned to the predicted structure of human UFSP1. The catalytic cysteine is highlighted by the box. (C) Schematic showing suggested model of the UFM1 pathway. In brief, precursor UFM1 is proteolytically activated through the removal of a C-terminal serine-cysteine peptide prior to sequential loading onto the E1, E2, and E3 conjugating enzymes. This culminates in modification of the ribosomal subunit RPL26. UFSPs act at several points in this pathway; (1) both UFSP1 and UFSP2 contribute to pro-UFM1 processing; (2) UFSP1 catalyzes the removal of UFM1 from UFC1, releasing UFC1 from a potentially autoinhibitory state; (3) UFSP2 catalyzes the removal of UFM1 from RPL26, preventing excess ribosome modification. ODR4 is essential for stabilizing UFSP2 and anchoring it at the ER membrane in proximity to the ribosome.

To address the biological significance of these findings, the proteomes of the different cell lines (*UFSP1*^*−/−*^, *UFSP2*^*−/−*^, and *UFSP1*^*−/−*^/*UFSP2*^*−/−*^ HEK293) were analyzed by data independent acquisition (DIA) quantitative proteomics. A total of 577 proteins passed the fold change and significance thresholds in at least one phenotype (Benjamini-Hochberg adjusted p < 0.05; log_2_ fold change > 1) (Figures 6C and S6C). Heatmap analysis (k-means method) revealed that distinct subsets of proteins are altered in UFSP1 and UFSP2 knockout cells (Figures 6C, S6D, and S6E). Gene ontology overlaps calculated using a hypergeometric distribution tool provided by the molecular signatures database (msigdb; https://www.gsea-msigdb.org/) suggest contributions of UFSP1 to small-molecule metabolism (Figure 6D). By contrast, in *UFSP2*^*−/−*^ HEK293 cells, processes including tissue development (VEGFA, FGFR1, BMP7, TIMP1, COL6A6) and lipid metabolism (LOX, ALOX5) were heavily represented, while *UFSP1*^*−/−*^/*UFSP2*^*−/−*^ cells exhibited features of both *UFSP1*^*−/−*^ and *UFSP2*^*−/−*^ knockout cell lines (Figures 6C, 6D, and S6C–S6E). Proteins subject to the most extreme changes (COL6A6, CDKN2A, RPL22L1) were common to all genotypes (Figure 6E). Although ER-linked proteins were represented, we observed no effect on canonical ER stress or UPR-linked pathways (Figure S7A). Immunoblot analysis of cell fractions confirms that UFSP1 is cytosolic in localization and, together with bioinformatic interpretation of UFSP2-ODR4 interactions, supports our conclusion that cellular localization is key to understanding the unique functionality observed in proteomes of *UFSP1*^*−/−*^ and *UFSP2*^*−/−*^ knockout cell lines (Figures 7A–7C).

## Discussion

Considering that 99 deubiquitinating enzymes have been identified in the analogous ubiquitin system (Lange et al., 2021), it seemed remarkable that the UFM1 pathway in humans could be reliant on only one enzyme. Together with the confounding observation of active UFMylation in *UFSP2*^*−/−*^ cell lines, it has been clear to many in the field that additional enzymes must exist to process precursor UFM1 into its mature counterpart (Witting and Mulder, 2021). Our study now reveals that the annotation of UFSP1 as inactive is mistaken, as we identify human UFSP1 to be translated from a non-canonical CTG start codon. We report that human UFSP1 is not only an active cysteine protease that is expressed in cells but also one with key functions in the UFM1 pathway. Overall, we define at least three contributions of UFSP enzymes. First, consistent with reports elsewhere, we find UFSP2 to be a key regulator of RPL26 modification. Second, UFSP1 acts to restrain levels of UFC1 modified with UFM1. Finally, in a partially redundant manner, both UFSP enzymes contribute to UFM1 precursor maturation and the maintenance of a cellular pool of mature UFM1 (Figure 7A).

One of two mechanisms may contribute to the different substrate activities of UFSP enzymes. First, the cellular expression levels of UFSP1 are very low, possibly limiting its ability to counter RPL26 UFMylation. Second, subcellular localization: the cytosolic localization of UFSP1 compared with the ER localization of UFSP2 makes it more likely for UFSP1 to be the UFM1-maturing enzyme. The transmembrane domain of ODR4 provides a means for UFSP2 to associate with the ER membrane, bringing it into proximity of the ribosome and UFM1 ligase machinery (Chen et al., 2014). Structure prediction of ODR4 using AlphaFold reveals it to adopt an MPN fold (Jumper et al., 2021). Interestingly, the crystal structure of Ufsp from *C*. *elegans* reveals the presence of an MPN fold in addition to the catalytic domain (Kim et al., 2018). It remains to be investigated whether ODR4 only mediates ER localization of UFSP2 or has additional roles to allosterically modulate the activity of UFSP2. Superposition of an AlphaFold-predicted complex of UFSP2-ODR4 reveals that UFSP1 lacks domains required for interaction with ODR4 (Figure 7B). Hence, UFSP1 is localized to the cytosol where it is unable to interact with UFMylated RPL26 at the endoplasmic reticulum.

We observe a striking accumulation of UFMylated UFC1 in *UFSP1*^*−/−*^ cells but not *UFSP2*^*−/−*^ cells. This suggests that UFC1 is constantly UFMylated in cells, and this modification is removed by UFSP1. Interestingly, previous studies have suggested regulation of E2 activity by covalent modification (Stewart et al., 2016). It may be that UFC1 UFMylation alters protein interactions with other UFM1-conjugating enzymes or putative substrates, either sterically or through the addition of a UFM1-interacting surface. For instance, the SUMO-specific E2 Ubc9 (UBE2I) is auto-SUMOylated, a modification that serves to attract proteins with SUMO-interacting motifs (Stewart et al., 2016). Of note, a UFM1 interacting motif has been described and may serve a similar purpose here (Padala et al., 2017; Kumar et al., 2021). Alternatively, auto-UFMylation may interfere with E2 catalytic activity. A recent case study of UBE2S automodification identified a lysine residue precisely five amino acids from the catalytic cysteine (K^+5^) (Liess et al., 2019). In thioester transfer assays, automodification at this site on UBE2S reduced the overall efficiency of ubiquitin transfer from the E1 by steric hindrance. Intriguingly, up to 25% of E2 enzymes possess a conserved lysine residue at +5 amino acids from the catalytic cysteine (Liess et al., 2019). Following a similar theme, monoubiquitylation of UBE2T at K86 (5 amino acids from the catalytic cysteine K91) is suggested to reduce E2 activity linked to the Fanconi anemia DNA repair pathway mediated by the E3 ubiquitin ligase FANCL (Machida et al., 2006). Our work identifies UFC1 to be modified on K122, a site located +6 residues from the catalytic C116. While further work is required, we speculate a similar inhibitory role for this UFC1 modification. These observations may reflect a common regulatory feature of E2 enzymes. If so, then UFSP1 may be the first documented instance of a protease acting to relieve E2 autoinhibition, eventually influencing the rate of overall UFMylation.

Hence, UFSP1 may act at two levels to activate UFMylation, firstly in UFM1 maturation and secondly as part of a regulatory loop to remove the inhibitory UFC1 modification. Given that *UFSP1* gene expression is remarkably low relative to other UFM1 pathway components, it is possible that under specific circumstances, *UFSP1* gene induction may function as an inducible ON-OFF switch or accelerator for ribosome UFMylation. Further studies will be required to assess whether this is the case and to assess the precise kinetics of UFMylation in cellular models with *UFSP1* overexpression or deletion. Moreover, studies examining whether UFSP1 contributes to other functions attributed to the UFM1 pathway, including regulation of the DNA damage response via histone H4 UFMylation or ER-phagy induced by metabolic stress, are likely to reveal novel insights (Qin et al., 2019; Liang et al., 2020). In particular, our proteomic analysis aligns with observations elsewhere that UFMylation may be integral to the development of extracellular matrix (Egunsola et al., 2017; Walczak et al., 2019).

A serendipitous outcome of our study is the correction of the long-held misconception of UFSP1 as an inactive peptidase homolog. This misunderstanding stems from the annotation of UFSP1 as the “inactive UFM1-specific protease-1” (HGNC annotation) and is based on a hypothetical interpretation of an N-terminal truncated version of UFSP1 that lacks catalytic residues when compared with UFSP2. Perhaps early studies characterizing UFSP1, performed in murine systems, were reliant on annotations that predate entry of the long isoform described here (Kang et al., 2007; Ha et al., 2008). Clearly, the naming of UFSP1 as the “inactive UFM1-specific protease-1” will require revision. Our study corrects the view that human UFSP1 is catalytically inactive, and in doing so has laid foundations for future investigation into the unique contributions of UFSP family proteases to ER and cellular homeostasis.

### Limitations of the study

A major substrate of UFSP1 appears to be UFC1, as UFMylated UFC1 modified at K122 accumulates in cells lacking UFSP1. Our work does not determine the cellular conditions that promote UFC1 modification at this potential autoinhibitory site. Furthermore, it will be important to establish the kinetics of this UFC1 modification in cells. Our investigation of UFSP1 and UFSP2 function in this study has employed constitutive knockout cell lines that may be subject to cellular adaptation. Future studies using acute depletion or inhibition of UFSPs will shed light on the dynamics and effect of rapid changes in UFMylation. Moreover, quantitative and temporal comparison of UFSP1 and UFSP2 activity on their respective substrates will inform the significance of this regulatory circuit.

## STAR★Methods

### Key resources table

**Table:** 

REAGENT or RESOURCE	SOURCE	IDENTIFIER
**Antibodies**		

Rabbit monoclonal anti-UFM1	Abcam	ab109305
Rabbit polyclonal anti-UCH-L1	CusaBio	CsB-PA004381
Rabbit monoclonal anti-UFSP2	Abcam	ab192597
Rabbit polyclonal anti-UFSP1	Sigma-Aldrich	HPA027099
Rabbit polyclonal anti-GFP	Abcam	ab290
Rabbit polyclonal anti-RPL26	Bethyl	A300-686A-M
Rabbit polyclonal anti-RPL26	Abcam	ab59567
Rabbit polyclonal anti-UBA5	Bethyl	A304-155A-T
Rabbit polyclonal anti-ERp72	CST	5033P
Rabbit polyclonal anti-GAPDH	CST	2118S
Mouse monoclonal anti-HA	MRC PPU Reagents and Services	Mouse Monoclonal 12CA5
Rabbit monoclonal anti-UFC1	Abcam	Ab189252
Rabbit monoclonal anti-UFC1	Abcam	Ab189251
Rabbit polyclonal anti-ODR4	Abcam	Ab121495

**Bacterial and virus strains**		

*Escherichia coli* (BL21)	This study	BL21
*Escherichia coli* (DH5)	MRC PPU Reagents and Services	DH5

**Chemicals, peptides, and recombinant proteins**		

Iodoacetemide (IAA)	Sigma Aldrich	I1149-5G
Dithiothreitol (DTT)	Formedium	DTT100
N-ehylmaleimide (NEM)	Sigma Aldrich	04259-5G
Ethylenediaminetetraacetic acid tetrasodium salt dihydrate (EDTA)	Sigma Aldrich	E5134-500G
Ethylene-bis(oxyethylenenitrilo)tetraacetic acid (EGTA)	Sigma Aldrich	E3889-100G
Tris	VWR Chemicals	103157P
Sodium Chloride	VWR Chemicals	27810.364
Glycine	VWR Chemicals	10119CU
Imidazole	Sigma Aldrich	I2399-500G
Glycerol Bidistilled 99.5%	VWR Chemicals	24388.320
Triethylammonium bicarbonate (TEABC)	Sigma Aldrich	T7408-100ML
TRIS(2-Carboxyethyl)Phosphine Hydrochloride (TCEP)	Apollo Scientific	BIT0122
Pierce Trypsin, MS Grade	Pierce	90058
Tween20	Sigma Aldrich	P2287-500mL
NP40	Sigma Aldrich	P1379-1L
BSAovine Serum Albumin (BSA)	Sigma Aldrich	A7906-100G
Ampicilin Sodium Salt	Formedium	AMP100
IPTG	Formedium	IPTG025
2xTY medium	MRC PPU Reagents and Services	2xTY
AEBSF	Apolloscientific	BIMB2003
Benzamidine Hydrochloride	Sigma Aldrich	434760
2-Mercaptoethanol	Sigma Aldrich	M6250-100ML
NuPAGE LDS-Sample Buffer	Invitrogen	NP0007
Recombinant proteins	MRC-PPU; Peter et al., 2022	https://www.biorxiv.org/content/10.1101/2022.01.31.478489v2

**Critical commercial assays**		

DNeasy Blood and Tissue kit	Qiagen	69504
NuPAGE 4–12% Bis-Tris Gel 12 well	Invitrogen	NP0322BOX
Stataclone blunt ended cloning kit	Agilent	240207
Qiagen Maxiprep kit	Qiagen	12162
Pierce BCA assay	ThermoFisher	23225

**Experimental models: Cell lines**		

Flp-in TREx HEK293 cells	Thermo-Fisher	R75007
*UFSP1*^*−/−*^ Flp-in TREx HEK293 cells	This study	CR1046 (MRC-PPU)
*UFSP2*^*−/−*^ Flp-in TREx HEK293 cells	This study	CR1046 (MRC-PPU)
*UFSP1*^*−/−*^/*UFSP2*^*−/−*^ Flp-in TREx HEK293 cells	This study	CR1046 (MRC-PPU)
*UFSP1*^*−/−*^ U2OS cells	This study	CR1046 (MRC-PPU)
*UFSP2*^*−/−*^ U2OS cells	This study	CR1046 (MRC-PPU)
*UFSP1*^*−/−*^/*UFSP2*^*−/−*^ U2OS cells	This study	CR1046 (MRC-PPU)
*UFSP1*^*−/−*^ HeLa cells	This study	CR1046 (MRC-PPU)
*UFSP2*^*−/−*^ HeLa cells	This study	CR1046 (MRC-PPU)
*UFSP1*^*−/−*^/*UFSP2*^*−/−*^ HeLa cells	This study	CR1046 (MRC-PPU)
*ODR4*^*−/−*^*/UFSP1*^*−/−*^ Flp-in TREx HEK293 cells	This study	CR768 (MRC-PPU)
*ODR4*^*−/−*^*/UFSP2*^*−/−*^ Flp-in TREx HEK293 cells	This study	CR768 (MRC-PPU)
*ODR4*^*−/−*^*/UFSP1*^*−/−*^/*UFSP2*^*−/−*^ Flp-in TREx HEK293 cells	This study	CR768 (MRC-PPU)

**Oligonucleotides**		

UFSP1 Genotyping primer (Forward; CGGAG CCGAAAGGAAGTGTTGG)	This study	Sigma Aldrich
UFSP1 Genotyping primer (Reverse; GCAAGA GGTGAGTGCAGCCTT)	This study	Sigma Aldrich
UFSP2 Genotyping primer (Forward; CCAGGA TCCTCAGTATTTTGCG)	This study	Sigma Aldrich
UFSP2 Genotyping primer (Reverse; AGATGA ACTGGTTTTACCTTCCT)	This study	Sigma Aldrich
ODR4 Genotyping primer (Forward; TATCCTC TCCCTTATCCCAGGTA)	This study	Sigma Aldrich
ODR4 Genotyping primer (Reverse; TTTTTCC CAATCCCTCTCCCTC)	This study	Sigma Aldrich
UFSP1 CRISPR single guide (GCCGGGACTG GATCGGCTGCG)	This study; MRC PPU Reagents and Services	DU69526
UFSP1 CRISPR guide RNA sense A (GTCTGC CTCGCTCACTTCGGA)	This study; MRC PPU Reagents and Services	DU69530
UFSP1 CRISPR guide RNA anti-sense A (GCC ACGCAGCCGATCCAGTCC)	This study; MRC PPU Reagents and Services	DU69532
UFSP2 CRISPR guide RNA sense (GATCATTG AAAGGGAAAGCGG)	This study; MRC PPU Reagents and Services	DU57251
UFSP2 CRISPR guide RNA anti-sense (GGGC GTTACAGCTGCCAGGG)	This study; MRC PPU Reagents and Services	DU57259
ODR4 CRISPR guide RNA sense (GCTTTCAA ACATAAATCTCCA)	This study; MRC PPU Reagents and Services	DU60768
ODR4 CRISPR guide RNA anti-sense (GAACA GTCTCTTCTACAATGT)	This study; MRC PPU Reagents and Services	DU60772

**Recombinant DNA**		

pet15b His C3 UFM1-GSGEGRG-GFP	This study; MRC PPU Reagents and Services	DU59553
pGEX6P1 UFSP1 (long)	This study; MRC PPU Reagents and Services	DU68653
pGEX6P1 UFSP1 (short)	This study; MRC PPU Reagents and Services	DU47927
pcDNA5 FRT-TO UFSP1 (long)	This study; MRC PPU Reagents and Services	DU68654
pcDNA5 FRT-TO UFSP1 (short)	This study; MRC PPU Reagents and Services	DU68627
pcDNA5 FRT-TO pro-UFM1(1-85)	This study; MRC PPU Reagents and Services	DU68742
pcDNA5 FRT-TO mature UFM1(1–83)	This study; MRC PPU Reagents and Services	DU59408
pBabeD P U6 UFSP1 ex1 KO Sense A	This study; MRC PPU Reagents and Services	DU69530
pX335 UFSP1 ex1 KO Antisense A	This study; MRC PPU Reagents and Services	DU69532
pX459 UFSP1 ex1 KO Single Guide G1	This study; MRC PPU Reagents and Services	DU69526
pX459 UFSP1 ex1 KO Single Guide G2	This study; MRC PPU Reagents and Services	DU69527
pX459 UFSP1 ex1 KO single Guide G1	This study; MRC PPU Reagents and Services	DU69526
pBabeD P U6 UFSP1 ex1 KO sense A	This study; MRC PPU Reagents and Services	DU69530
pX335 UFSP1 ex1 KO Antisense A	This study; MRC PPU Reagents and Services	DU69532
pBabeD P U6 UFSP2 ex5 KO sense	This study; MRC PPU Reagents and Services	DU57251
pX335 UFSP2 ex5 KO Antisense	This study; MRC PPU Reagents and Services	DU57259
pBabeD P U6 ODR4 ex2 KO Sense A	This study; MRC PPU Reagents and Services	DU60768
pX335 ODR4 ex2 KO Antisense A	This study; MRC PPU Reagents and Services	DU60772
pGEX6P1-UFC1	This study; MRC PPU Reagents and Services	DU73281
pET15b-6xHis-3C-UBA5	This study; MRC PPU Reagents and Services	DU32106
pET15b-6xHis-TEV-UFM1	This study; MRC PPU Reagents and Services	DU73256

**Software and algorithms**		

Adobe Illustrator	Adobe	https://www.adobe.com/uk/
RStudio	RStudio	https://rstudio.com/
Muscle	European Bioinformatics Institute (EBI)	https://www.ebi.ac.uk/Tools/msa/muscle/
Complex heatmap	R-package (CRAN)	https://www.bioconductor.org/packages/release/bioc/html/ComplexHeatmap.html
Consurf	Tel Aviv University	https://consurf.tau.ac.il/
ChimeraX	University of California San Francisco (UCSC)	https://www.cgl.ucsf.edu/chimerax/
Jalview	Barton, GJ (University of Dundee)	https://www.jalview.org/

**Other**		

*MEROPS* database of peptidases and peptidase inhibitors	European Bioinformatics Institute (EBI)	https://www.ebi.ac.uk/merops/
Genotype-Gene expression project (GTEx)	Broad Institute	https://www.gtexportal.org/home/
DIANN1.8	Demichev et al. (2020)	https://www.nature.com/articles/s41592-019-0638-x
Riboseq data resource (GWIPS-VIZ).	GWIPS-VIZ	https://gwips.ucc.ie/
FactoExtra	R-package (CRAN)	https://cran.r-project.org/web/packages/factoextra/index.html

### Resource availability

#### Lead contact

Further information and requests for resources and reagents should be directed to and will be fulfilled by the lead contact, Yogesh Kulathu (y.kulathu@dundee.ac.uk).

#### Materials availability

Plasmids and cell lines are available upon request to the study lead author listed above. Identifier codes for plasmids and cell lines are included in the key resources table. All cell lines used in this study are maintained in a dedicated cell bank and are traceable by Cell line name, Clone number, and CRISPR project ID. The authors declare no restriction on the use of materials detailed herein.

### Experimental model and subject details

Cell lines were cultured in DMEM (GIBCO) supplemented with 10% v/v Fetal Bovine Serum (FBS), 50mg/ml Penicillin Streptomycin, and 2mM L-Glutamine. Cell cultures were maintained in a 5% CO_2_ incubator in a humidified environment and routinely checked for mycoplasma. Cell lines used in this study include U2OS, HeLa, HEK293, and commercially available Flp-In T-REx HEK293 cells (Invitrogen; R78007). Cell lines were sourced from a dedicated facility at MRC-PPU core services.

### Method details

#### Cell fractionation

HEK293T cells (10 confluent plates) were collected in phosphate-buffered saline/PBS (Gibco; 14190-094) supplemented with 1mM EDTA and 1mM EGTA. Cells were washed once in PBS, resuspended in ice-cold cracking buffer (50mM Tris pH7.5, 1mM DTT, 0.1mM EDTA, 0.1mM EGTA), and incubated on ice for 15 minutes before lysis by mechanical stress (>20 sequential passes through 21-23-gauge needles). The lysate was cleared by centrifugation (17000 x g for 5 minutes), passed through a 25mm/45μm polyethersulfone filter (Sigma Aldrich; WHA68962504), and de-salted into a desalting buffer (30mM MOPS pH7.0, 5% glycerol, 1mM DTT, 0.015% Brij 35) on a Sephadex G25 column using an Akta Pure Fast Protein Liquid Chromatography (FPLC) device. The lysate was next applied to a 1mL Heparin HiTrap column (GE-Healthcare Life Sciences, now Cytiva) with elution on an increasing salt gradient into 1mL fractions (Greiner bio-one 96 well blocks; 780270). A salt gradient was introduced using a high salt buffer (30mM MOPS pH7.0, 1.2M NaCl, 5% glycerol, 1mM DTT, 0.015% Brij 35). Heparin column flow-through was next passed through a Source Q HR 5/5 column into 30mM Tris-HCl pH8.2, 5% Glycerol, 1mM DTT, and 0.015% Brij 35 with elution on a salt gradient (High salt buffer supplemented with 1.0M NaCl). SourceQ fractions (1mL volume) were eluted into a 96 well block at 1mL intervals. Heparin and SourceQ binding fractions were immediately snap-frozen in liquid nitrogen and stored at −80°C until use. For mass spectrometry, fractions were washed/buffer exchanged in an Amicon ultra centrifugal concentrator (Millipore; UFC500396) with 2mL 30mM Tris-HCl pH 7.5 containing 1mM TCEP, followed by 2mL 50mM triethylammonium bicarbonate buffer (Sigma Aldrich T7408-100mL) containing 1mM TCEP. Samples were then concentrated to approximately 100μL volume and alkylated with 40mM IAA (Sigma Aldrich; I1149-5G) for 3 hours in the dark at room temperature. Samples were next reduced by adding 2mM DTT and incubating at room temperature for a further 15 minutes (Formedium; DTT100). After overnight digestion with 10μg/ml mass-spectrometry grade Trypsin (Pierce; 90057), samples were submitted to the MRC-PPU mass-spectrometry facility for analysis.

#### Western blotting

To prepare samples a 3:4 dilution was made in NuPAGE LDS sample buffer (ThermoFisher; NP007) supplemented with 10% v/v β-mercaptoethanol. Samples were heated to 95°C for 5 minutes before gel loading. Gel electrophoresis was performed using an XCell *SureLock* electrophoresis tank (ThermoFisher; EI0001) with 4–12% Bis-Tris NuPAGE pre-cast 12, 15, and 26 well gels (Invitrogen; NP0322, NP0323, and WG1403BX10). Protein was transferred to a 45μm nitrocellulose membrane (Amersham; 10600002) at 90V for 90minutes in a Mini Trans-Blot Cell (Biorad;). Membranes were blocked for one hour in 5% Bovine Serum Albumin (Sigma Aldrich; A7906-100G)) dissolved in TBST. Primary antibodies were diluted 1:1000 in 5%BSA TBST and incubated overnight with shaking at 4°C. Membranes were washed three times for 10 minutes per wash in TBST. Membranes were next incubated with fluorescent secondary antibody (IR800) diluted 1:20,000 in TBS containing 5%BSA, 0.1% Tween for 30 minutes at RT with shaking. After washing in TBST for a further 30 minutes (3 × 10-minute washes) membranes were visualized on a Lycor Odyssey CLx.

#### Cell lysates

To generate cell lysates for RPL26 immunoblots, one near-confluent 15cm plate of HEK293 cells was gently collected in 0.5mM EDTA/0.5mM EGTA and placed on ice. Cells were washed once in ice-cold PBS and resuspended in lysis buffer (1%NP40, 50mM Tris-HCl pH7.5, 150mM NaCl) supplemented with a protease inhibitor cocktail (1mM benzamidine, 1mM AEBSF, Protease inhibitor cocktail (Roche; 48679800)). Lysates were cleared by centrifugation (20,000 x g, 5 minutes) and mixed with LDS sample buffer as before. For enzymatic assays, cell lysates were generated in the absence of protease inhibitors as described in the cell fractionation procedure above. For chemical induction of UFMylation cells were treated with 200nM Anisomycin dissolved in DMSO for 20 minutes before harvesting.

#### Cell membrane cytosolic and membrane fractionation

HEK293 or HeLa cells were washed once in PBS, collected in ice-cold PBS, and pelleted by centrifugation at 1000 x g. Cell pellets (∼1 × 10^7^) were resuspended in 1 mL of 0.02% w/v digitonin, 50 mM HEPES pH 7.5, 150 mM NaCl, 2 mM CaCl_2_, and 1 x protease inhibitor cocktail tablet EDTA-free. Lysates were incubated on ice for 10 min and centrifuged at 17000 x g for 10 min at 4°C. The supernatant was transferred to a new Eppendorf tube (cytoplasmic extract). The remaining pellet was washed with 1 mL PBS and resuspended in 1 mL of 1% Triton X- 100, 50 mM HEPES pH 7.5, 150 mM NaCl, and 1x protease inhibitor cocktail tablet EDTA-free. Lysates were incubated on ice for 10 minutes and centrifuged at 17000 × g for 10 min at 4°C. The supernatant was transferred to a new Eppendorf tube (membrane extract). The remaining pellet was washed with 1 mL PBS and sonicated in 1 mL of 1% SDS, 25 mM Tris pH 8, 150 mM NaCl, 2.5 mM EDTA, and 1x protease inhibitor cocktail tablet EDTA-free (nuclear extract). Equal volumes of the collected fractions were resolved by SDS-PAGE and subjected to immunoblot analysis.

#### Cloning

Pro-UFM1 (NP_057701.1) was cloned in frame with an N-terminal GFP tag and intervening short peptide linker (GSGEGRG) into the pcDNA5/FRT/TO bacterial expression vector (Invitrogen; V652020). A C-terminal histidine tag (Hisx6) with a C3 protease site facilitated the generation of native protein after purification with Ni^2+^/NTA affinity beads. UFSP1 short and long variant isoforms were cloned into the pGEX6P1 vector for bacterial expression. UFSP2 (modified with a stabilizing R136A mutation) was cloned into the petDuet (His6-TEV-UFSP2; DU59927) bacterial expression vector in frame with an N-terminal 6xHis-tag. A full list of cDNA constructs is included in the key resources table.

#### Recombinant protein expression and purification

Recombinant GST-3C-tagged UCH-L1, UCH-L3, UCHL-5, and BAP were obtained from MRC-PPU Reagents and Services (https://mrcppureagents.dundee.ac.uk/).

UFSP1 short (Q6NVU6) and long (A0A5F9ZGY7) isoforms and UFC1 (DU47927, DU68653, and DU73281, respectively) were expressed with GST-3C tags and purified using Glutathione S-transferase (GST) affinity purification. Briefly, expression constructs were transformed into *E*.*coli* BL21(DE3) competent cells, and expression of the recombinant protein was induced with 0.25 mM IPTG overnight (∼16 hours) at 18°C. Cells were sedimented by centrifugation, resuspended in lysis buffer (50 mM Tris-HCl pH 8.0, 300 mM NaCl, 2 mM DTT), and supplemented with a protease inhibitor cocktail (1 mM benzamidine, 1 mM AEBSF, 1x protease inhibitor cocktail (Roche; 48679800)), and lysed by Ultra sonification. The lysate was cleared by ultracentrifugation at 30,000 x g for 30 minutes and mixed with Glutathione Sepharose 4B beads for approximately 1.5 hours at 4°C. Beads were washed with high salt wash buffer (50 mM Tris-HCl pH 8.0, 500 mM NaCl, 2mM DTT) and low salt wash buffer (50 mM Tris-HCl pH 8.0, 150mM NaCl, 2 mM DTT, 10% Glycerol). Protein was eluted by incubation with 0.1 mg 3C protease (MRC-PPU Reagents and Services) in 10 mL low salt wash buffer overnight at 4°C. The cleaved protein was further purified on a Superdex-75 gel filtration column. The purified protein was concentrated, aliquoted, snap-frozen in liquid nitrogen, and stored at −80°C until further use.

##### UBA5, UFM1, UFM1-GFP fusion proteins

pET15b-6xHis-3C-UBA5 (DU32106) and pET15b-6xHis-TEV-UFM1 (DU73256) and pET15b-6xHis-3C-UFM1-GSGEGR-GFP (DU59553) were transformed into *E*.*coli* BL21(DE3) competent cells. Expression of the recombinant protein and cell lysis was performed as described above. The lysate was cleared by ultracentrifugation at 30,000 x g for 30 minutes, and then mixed with Ni^2+^ NTA beads in binding buffer (25 mM Tris pH 8.0, 300 mM NaCl, 10 mM imidazole, 1 mM DTT). Beads were then washed with 30-bed volumes of wash buffer (25 mM Tris pH 8.0, 300 mM NaCl, 20 mM imidazole, 2 mM DTT). UBA5 and UFM1 were eluted with elution buffer (50 mM Tris pH 8.0, 200 mM NaCl, 300 mM imidazole, 1 mM DTT). UFM1 and UFM1-GFP were eluted by incubation with TEV and 3C protease (MRC-PPU Reagents and Services), respectively, overnight at 4°C. Proteins were further purified on a Superdex-200 HiLoad ™ 16/600pg (UBA5, UFM1-GFP) or Superdex-75 HiLoad ™ 16/600pg (UFM1) gel filtration column. Peak fractions were concentrated to 2–16 mg/mL, snap-frozen in liquid nitrogen, and stored at −80°C until use.

##### Generation of UBA5-UFM1 and UFC1-UFM1

UFMylated (UBA5-UFM1) was generated by incubating UBA5 (0.01 mM) with UFM1 (0.01 mM) in reaction buffer (50 mM Tris pH 8.0, 200 mM NaCl, 5 mM ATP, 5 mM MgAc) for 17 hours at 23°C. UFC1-UFM1 was generated by incubating UFC1 (0.05 mM) with UBA5 (2 μM) and UFM1 (0.05 mM) in reaction buffer for 4 h at 37°C. Reaction products were purified on Superdex-200 HiLoad ™ 16/600pg (UBA5-UFM1) and Superdex-75 HiLoad ™ 16/600pg (UFC1-UFM1). Peak fractions were concentrated, snap-frozen in liquid nitrogen and stored at −80°C until use.

#### GFP cleavage/DUB assay

Whole-cell lysates (WCL) were extracted from *UFSP2*^*−/−*^ HEK293 cells by mechanical lysis (syringe), thiol proteases ‘activated’ by addition of 10mM Dithiothreitol (DTT) (Kulathu et al., 2013; Lee et al., 2013). and incubated with the UFM1-GFP fusion protein. Cell fractions and/or recombinant enzymes were pre-activated on ice in Activation Buffer (50mM Tris-HCl pH7.5, 50mM NaCl) supplemented with 10mM freshly prepared DTT. The activated enzyme was next incubated with 5ug (3μM) recombinant UFM1-GFP fusion protein for 3 hours at 37°C. Cleavage of the GFP tag was analyzed by Coomassie stain and/or immunoblot analysis. For assays involving chemical inhibitors, Iodoacetmide (Sigma-Aldrich; I1149-5G) or N-ethylmaleimide (Sigma-Aldrich; 04259-5G) were added to the enzyme for 1 hour at room temperature in the dark before mixing with recombinant UFM1-GFP. All enzymatic reactions were completed in ultra-pure distilled water (Millipore QPOD; ZMQSP0D01).

#### CRISPR-Cas9

CRISPR guide RNAs were designed with support from T. MacCartney at MRCPPU Reagents and Services. CRISPR sense and anti-sense guides were cloned into pX335 (Addgene plasmid 42335; Feng Zhang lab; Massachusetts Institute of Technology) and pBABED puro U6 (DU48788) plasmids respectively. The pX335 construct contains a chicken β–actin promoter-driven expression cassette for Cas9. In a separate strategy, single guide RNAs were cloned into the px459 vector (Addgene; 48139). Full details of guide-RNAs, frameshift mutations, and relevant sequencing data are included in the supplementary figures and key resources table. Procedures are described elsewhere (Ran et al., 2013a, 2013b): briefly, 1–2 million cells were seeded into a 10cm dish in antibiotic-free Dulbecco’s Modified Eagle Medium (DMEM) and transfected with 1μg plasmid DNA using Lipofectamine 2000 (Invitrogen; 1168019) according to the manufacturer’s instructions. Cells were selected in 2μg/ml puromycin for 24 hours followed by a 24-hour recovery period in pre-conditioned media. Cells were plated at clonal dilution (0.7 cells/well) or submitted for single-cell sorting, expanded, and screened by sequencing and/or immunoblot analysis.

#### Sequencing

For UFSP1, UFSP2, and ODR4 knockout clones, a ∼1-1.5Kb fragment that included guide-RNA target sites was PCR amplified using Q5 High-Fidelity DNA Polymerase (NEB; M0491). Primers were designed using the NCBI Primer Blast tool and are documented in the key resources table. PCR products were purified by spin column (QIAGEN;28104) and cloned into a plasmid vector using the StrataClone blunt PCR cloning kit (Agilent; 240207). Colonies were selected and grown in 4mL 2xTY media supplemented with Ampicillin (10μg/ml). Plasmid DNA was extracted using the QIAprep Spin Miniprep kit (QIAGEN; 27104) and submitted for sequencing at the MRC PPU DNA sequencing and services division. Mutations were aligned to the Hg38 assembly (UCSC genome browser) using ClustalW (European Bioinformatics Institute; Muscle). Primers are detailed in the key resources table.

#### Data visualization and software

Western blots were processed in ImageStudio Lite (Licor) and arranged in Adobe illustrator. Original Graphics and cartoons were developed in Adobe Illustrator. Data filtering and analysis of public resources (MEROPS, GTEx) were completed in RStudio. Heatmaps were generated using the Complex Heatmap R-package (ComplexHeatmap) in R studio and clustered using default parameters (Euclidean method). Protein structures were visualized in ChimeraX. For the heatmap in Figure 6C, a k-means clustering approach (n = 3) was applied following statistical analysis using the Elbow method. Proteins within clusters are grouped by the Euclidean method.

#### External resources

Protein homology was analyzed using Consurf (Tel Aviv University; https://consurf.tau.ac.il/) and visualized in Pymol (Educational license V2) by Schrodinger (https://pymol.org/2/). Mass-spectrometry results were aligned with the MEROPS database (European bioinformatics Institute; https://www.ebi.ac.uk/merops/) to discover novel peptidases. Gene expression data was downloaded from the Genotype-Gene expression project (GTEx) (Broad Institute; https://www.gtexportal.org/home/). For protein copy number analysis across 32 human tissues, the dataset PXD016999 from ProteomeXchange (https://doi.org/10.1016/j.cell.2020.08.036) (Jiang et al., 2020) was reanalyzed using MaxQuant 2.0.3.1.6.

#### Multiple sequence alignment

fasta files corresponding to the amino acid sequence of human UFSP1 (Q6NVU6; A0A5F9ZGY7) and UFSP2 (H0Y9B0; H0YA18; D6RA67; Q9NUQ7) protein-coding transcripts were downloaded from Uniprot with reference to Ensembl annotation. Analogous sequences from other species were obtained with reference to the NCBI Homologene resource. Sequence alignment was performed using the ClustalW algorithm in Muscle (European Bioinformatics Institute). The ClustalW output was visualized in Jalview and edited in Adobe Illustrator.

#### Data Independent Acquisition (DIA) proteomics

For each sample, a confluent 15cm plate of HEK293 cells was resuspended in ice-cold PBS (1mM EDTA/1mM EGTA), pelleted by centrifugation, and immediately lysed by addition of SDS-lysis buffer (5% SDS, 50mM TEAB pH8.5). Lysates were boiled for 5 minutes at 95°C followed by sonication using a Diagenode Biorupter at high energy for 10 cycles (30sec ON, 30sec OFF). Lysates were cleared by centrifugation at 20,000 x g for 20 minutes and quantified by BCA assay (Pierce; 23225). 200μg protein was prepared as follows; TCEP stock solution (100mM TCEP, 300mM TEABC) was added to a final concentration of 10mM TCEP (1:10), and samples were incubated at 60°C for 30 minutes. Samples were rested at room temperature and freshly prepared iodoacetamide (IAA) added to 40mM final concentration. After 30 minutes at room temperature shielded from light and with gentle agitation, samples were acidified by the addition of mass-spectrometry grade 12% phosphoric acid to a final concentration of 1.2% (1:10). Sample ‘clean-up’ was completed using S-trap micro-columns with overnight on-column digestion using 13μg trypsin per 200μg of protein input. Eluted peptides were lyophilized by speed-vacuum and submitted to the MRC-PPU core mass-spectrometry facility. For differential expression analysis data were processed using LIMMA. Data was analyzed in Dia-nn 1.8 (Demichev et al., 2020; Steger et al., 2021). Selected MSMS spectra of VG-modified peptides were annotated using IPSA.

### Quantification and statistical analyses

Statistical details are included in the figure legends. All experiments shown are representative of at least three independent experiments. Observations of CRISPR knockout cell lines include multiple biological replicates (independently isolated clones with different mutations) as described in the figure legends. For proteomics data analysis, three technical replicates (three plates of the same CRISPR clone) were processed in parallel. For analysis of proteomics data, we considered a Benjamini & Hochberg adjusted p-value of <0.05 as significant. An arbitrary Log_2_ fold cut-off value of >1 was applied to focus the analysis on proteins with the most robust change. Gene Ontology enrichments were calculated using a hypergeometric tool (msigdb) with a p-value of less than 0.05 considered significant. For heatmap analysis, k-means clustering was performed using R base functions in Rstudio. To determine the appropriate number of clusters the elbow statistic was applied using the FactoExtra R-package. Visualization and euclidean clustering of proteins within k-means clusters was performed using the Complex heatmap package. R-packages are detailed in the key resources table.

## Data Availability

• The published article includes all datasets generated and analyzed for this study. Original western blot images and analyzed proteomics data are available on request and will be fulfilled by the lead contact (y.kulathu@dundee.ac.uk). The mass spectrometry proteomics data have been deposited to the ProteomeXchange Consortium via the PRIDE partner repository with the dataset identifier PXD035142 (http://www.proteomexchange.org/). • No original code was used to perform the analysis. The code required for the analysis is accessible in the user information for the appropriate R package. All R-packages used are listed in the key resources table. • Any additional information required to reanalyze the data reported in this paper is available from the lead contact upon request.

## Supplemental information

Document S1. Figures S1–S7

Document S2. Article plus supplemental information
