# Supplementary material for: Human UFSP1 is an active protease that regulates UFM1 maturation and UFMylation
Source: Cell Rep. 2022 Aug 3;40(5):111168. doi: 10.1016/j.celrep.2022.111168 (PMC9638016; doi:10.1016/j.celrep.2022.111168)
Supplement: Document S1. Figures S1–S7 [file mmc1.pdf]

Cell Reports, Volume *40*

Supplemental information

Human UFSP1 is an active protease that regulates UFM1 maturation and UFMylation

David Millrine, Thomas Cummings, Stephen P. Matthews, Joshua J. Peter, Helge M. Magnussen, Sven M. Lange, Thomas Macartney, Frederic Lamoliatte, Axel Knebel, and Yogesh Kulathu

Supplementary Material

Figure-S1

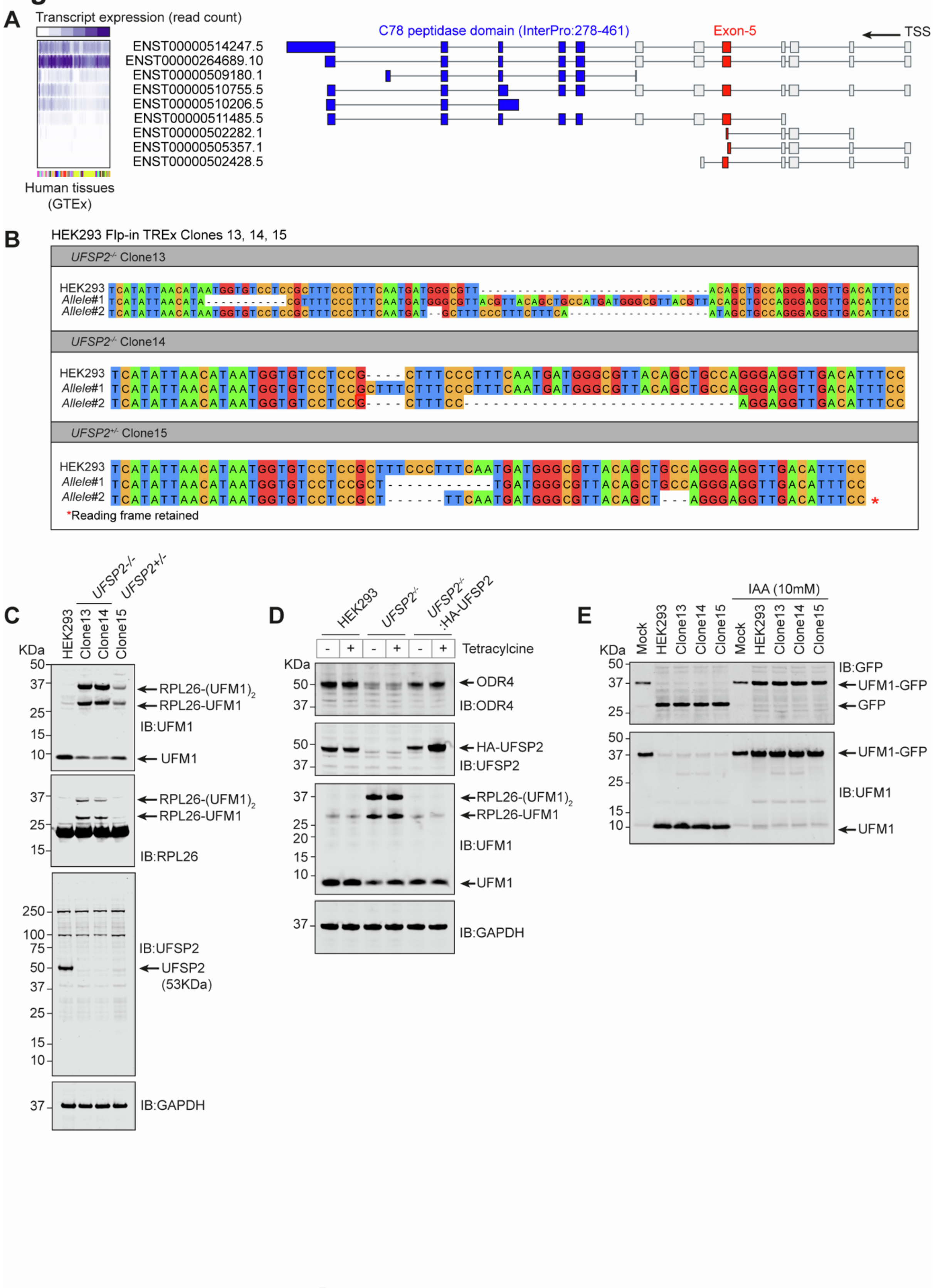

Figure-S1. Targeted knockout of UFSP2. Related to Figure 1. (A) Schematic of protein coding transcripts of UFSP2 as annotated by the GTEx resource (Broad

Institute). Figure was downloaded from the GTEx transcript browser and edited in Adobe Illustrator. Regions coding for the C78 protease domain and CRISPR gRNA target region are shown in blue and red respectively. Gene expression data (read count) is condensed and shown left – consult https://gtexportal.org/home/ for full data. **(B)** Sequencing analyses for CRISPR clones designated 13, 14, and 15. Sequences are representative of at least 8 bacterial colonies selected for miniprep and sequencing. Mutations were determined by multiple sequence alignment (ClustalW) to the Hg38 reference genome. Note that Clone 15 retains an active reading frame in one allele. Excerpt from the UCSC genome browser is shown top for reference **(C)** Immunoblot analysis of cell lysate from all clones. **(D)** Rescue of *UFSP2*$^{-/-}$ cell line phenotype by stable transfection of UFSP2 expressing cDNA. The HEK293 Flp-in TREx cell line was transiently transfected with pcDNA5 expressing HA-tagged UFSP2 and subject to antibiotic selection. **(E)** Cleavage assay using cell lysates derived from all clones. Where indicated, lysates have been pre-treated with Iodoacetamide (IAA) for one hour at room temperature in the dark. Experiments in C-D are representative of more than three experiments.

Figure-S2

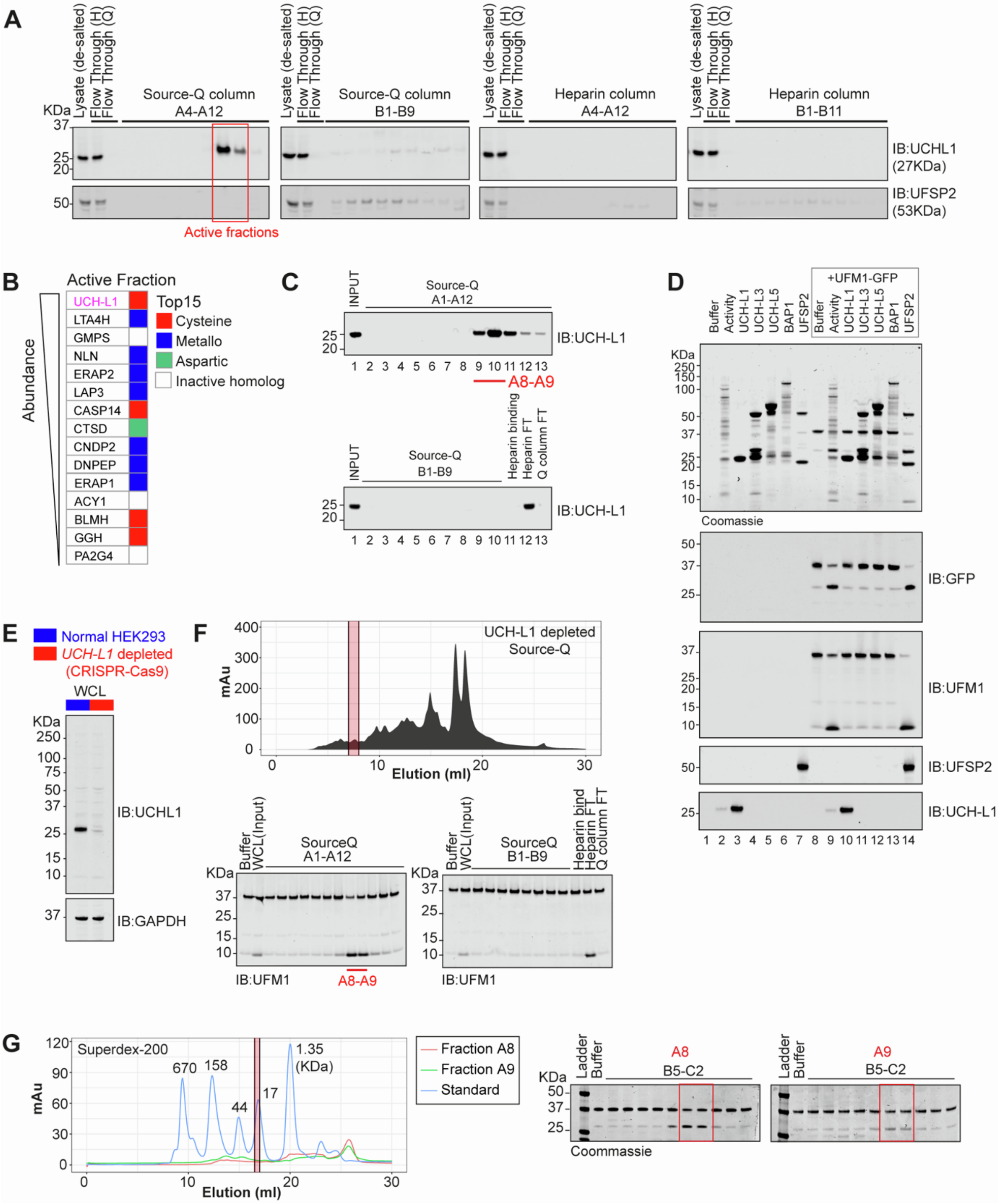

Figure-S2. UCH-L1 depletion and identification of long isoform UFSP1. Related to Figure 2. UCH-L1 was identified as a top hit in our initial screens. Speculatively, we considered that UCH-L1 may obstruct detection of the novel peptidase either by sequestration of column volume or through the saturation of mass-spectrometry detection. Therefore, CRISPR-Cas9 was employed to deplete UCH-L1

expression in HEK293 cells prior to the fractionation procedure. Data presented in Fig-2D-E are from experiments using UCH-L1 depleted cells. **(A)** Representative blotting of fractions eluted from Heparin and Source-Q columns showing UFSP2 distribution in relation to UCH-L1. Red boxes highlight the active fractions. **(B)** LC/MS analysis of active fractions derived from HEK293 cells showing UCH-L1 as the most frequently identified protease. Only the top 15 proteases are shown. **(C)** Immunoblot analysis showing distribution of UCH-L1 in fractions. Experiment is same as shown Fig-2B. **(D)** In vitro cleavage assay in which the UFM1-GFP probe incubated with UCH-L1 and related DUBs. **(E)** Immunoblot analysis of UCH-L1 depleted cell lines generated through transfection of *UCHL1* targeting CRISPR-Cas9 constructs. Small amounts of UCH-L1 are detected by LC/MS shown Fig-2D. **(F)** (top) Akta chromatogram showing fractionation of UCH-L1 depleted HEK293 cells (HiLoad® 16/600) (below) *In vitro* cleavage assay incubating fractions with UFM1-GFP probe. **(G)** fraction A8 and A9 in (E) were subject to further clean-up on a Superdex-200 pg column. Activity in fractions was confirmed by cleavage assay (right) prior to analysis by LC/MS. Resulting data is shown in Fig-2D.

Figure-S3

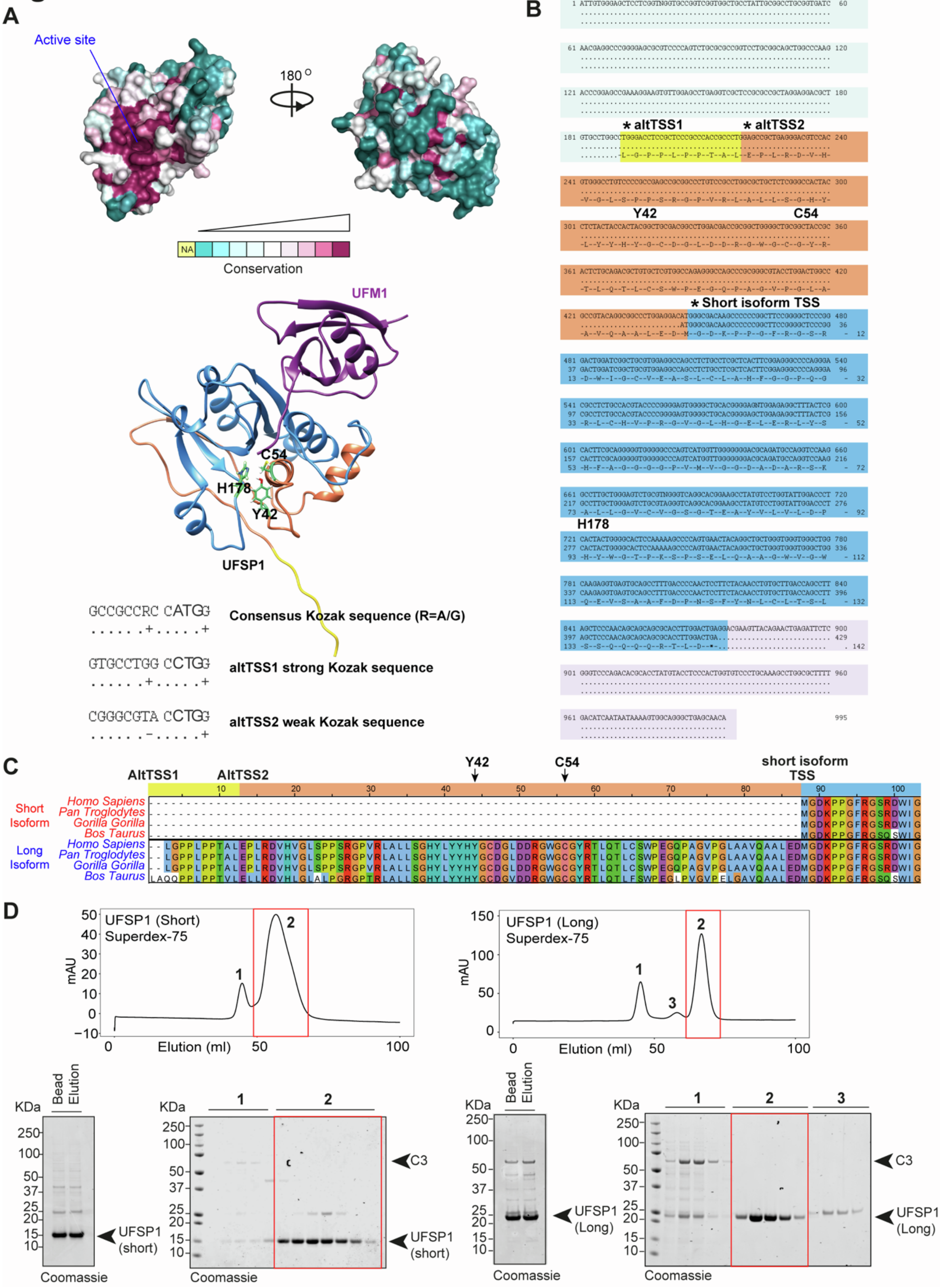

A
Active site
180°
NA
Conservation
UFM1
C54
H178
Y42
UFSP1
GCCGCCRC CATGG
Consensus Kozak sequence (R=A/G)
GTGCCTGG CCTGG
altTSS1 strong Kozak sequence
CGGGCGTA CCTGG
altTSS2 weak Kozak sequence
B
* altTSS1
* altTSS2
Y42
C54
* Short isoform TSS
H178
C
AltTSS1
AltTSS2
Y42
C54
short isoform TSS
Short Isoform
Homo Sapiens
Pan Troglodytes
Gorilla Gorilla
Bos Taurus
Long Isoform
D
UFSP1 (Short)
Superdex-75
UFSP1 (Long)
Superdex-75
mAU
Elution (ml)
KDa
Bead
Elution
C3
UFSP1 (short)
UFSP1 (Long)
Coomassie

Figure-S3. UFSP1 expression and purification. Related to Figure 3. (A) Evolutionary conservation of amino acids in UFSP1 depicted on a surface representation of crystal structure of murine UFSP1 using consurf (PDB ID: 2Z84). (below) Alphafold prediction of UFSP1 in complex with UFM1. Coloring corresponds to sequence analysis shown in (B). **(B)** Bioinformatic analysis of UFSP1 transcripts. Highlighted are key features including predicted transcription start sites and catalytic residues. **(C)** Cross-species multiple sequence alignment highlighting long isoform unique residues **(D)** Gel-filtration clean-up of human recombinant short (Q6NVU6) and long (A0A5F9ZGY7) UFSP1 isoforms. Raw elution from beads, after immunoprecipitation and 3C cleavage, is shown left for reference. Duplicate lanes are technical replicates.

Figure-S4

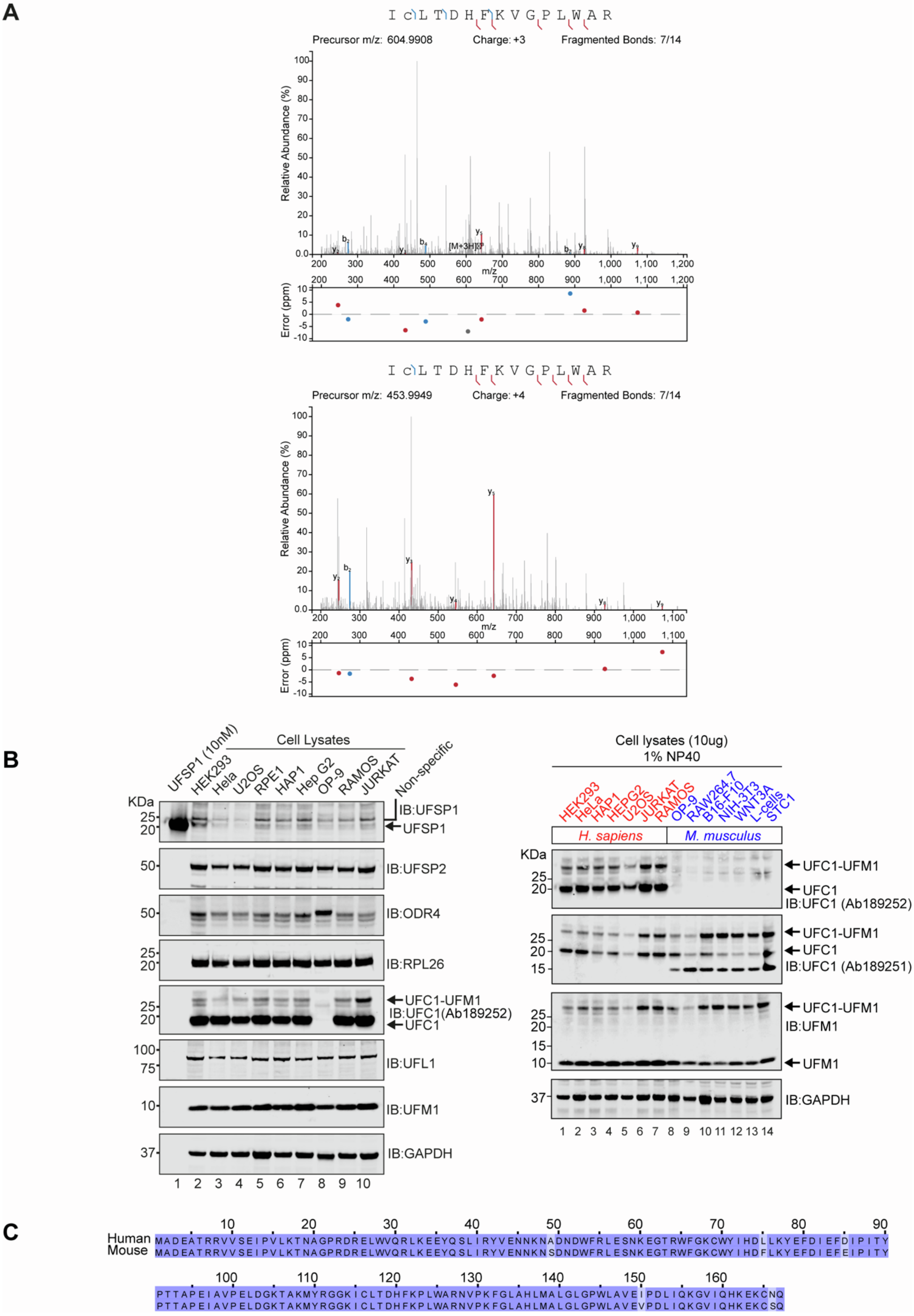
A
I C L T D H F K V G P L W A R
Precursor m/z: 604.9908
Charge: +3
Fragmented Bonds: 7/14
Relative Abundance (%)
m/z
Error (ppm)
Precursor m/z: 453.9949
Charge: +4
Fragmented Bonds: 7/14
B
Cell Lysates
UFSP1 (10nM)
HEK293
Hela
U2OS
RPE1
HAP1
Hep G2
OP-9
RAMOS
JURKAT
Non-specific
KDa
IB:UFSP1
UFSP1
IB:UFSP2
IB:ODR4
IB:RPL26
UFC1-UFM1
IB:UFC1(Ab189252)
UFC1
IB:UFL1
IB:UFM1
IB:GAPDH
Cell lysates (10ug)
1% NP40
HEK293
HeLa
HAP1
HEPG2
U2OS
JURKAT
RAMOS
OP-9
RAW264.7
B16-F10
NIH-3T3
WNT3A
L-cells
STC1
H. sapiens
M. musculus
IB:UFC1 (Ab189252)
IB:UFC1 (Ab189251)
UFM1
C
Human
Mouse

Figure-S4. Modification of UFC1 Lysine 122 with UFM1. Related to Figure 4. (A) MSMS spectra showing UFC1 K122 with the expected UFM1 VG motif tryptic remnant resulting from UFMylation. Spectra were generated from the analysis of DIA proteomics using Spectronaut 15. Selected MSMS spectra of VG modified peptides were annotated using IPSA (http://www.interactivepeptidespectralannotator.com)(Brademan *et al.*, 2019). **(B)** SDS-PAGE analysis of cell lysates from the indicated cell lines. Immunoblotting with anti-UFC1 (Abcam; Ab189252) failed to detect murine UFC1 (left). (right) Immunoblotting with anti-UFC1 (Abcam; Ab189251) confirms UFC1 expression in murine cell lines and reveals a short isoform variant unique to murine cell lines. **(C)** Multiple sequence alignment of annotated murine and human UFC1 isoforms.

Figure-S5

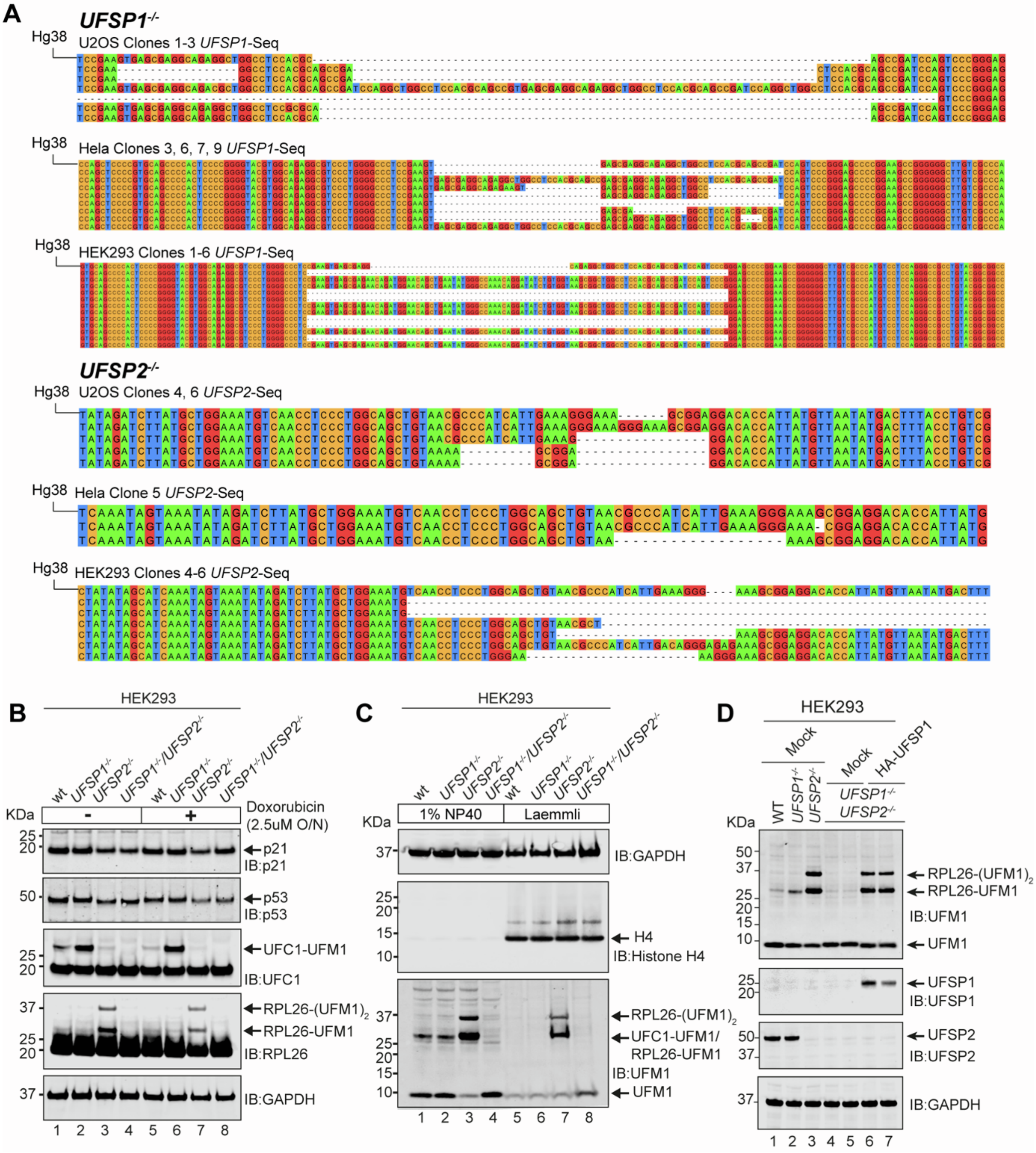

Figure-S5. UFSP1 function in ribosome UFMylation. Related to Figure 5. (A) Representative sequencing data for clones shown in Fig-5A. Each clone was sequenced a total of 8 times and aligned with the reference genome (Hg38) using Muscle (European Bioinformatics Institute). Shown are two sequencing traces representative of each allele, at the approximate location of Cas9-induced mutation. Mutations were determined by multiple sequence alignment (ClustalW) of sequencing traces to the Hg38 reference genome. **(B-C)** SDS-PAGE analysis of proteins modified

by UFM1. No effect was observed on P53 or Histone H4 under resting conditions. **(D)** Rescue of loss-of-function phenotype by UFSP1 over-expression. *UFSP1*$^{-/-}$/*UFSP2*$^{-/-}$ HEK293 cells were transiently transfected as indicated. The anti-UFM1 western blot is reproduced in Fig-5D.

Figure-S6

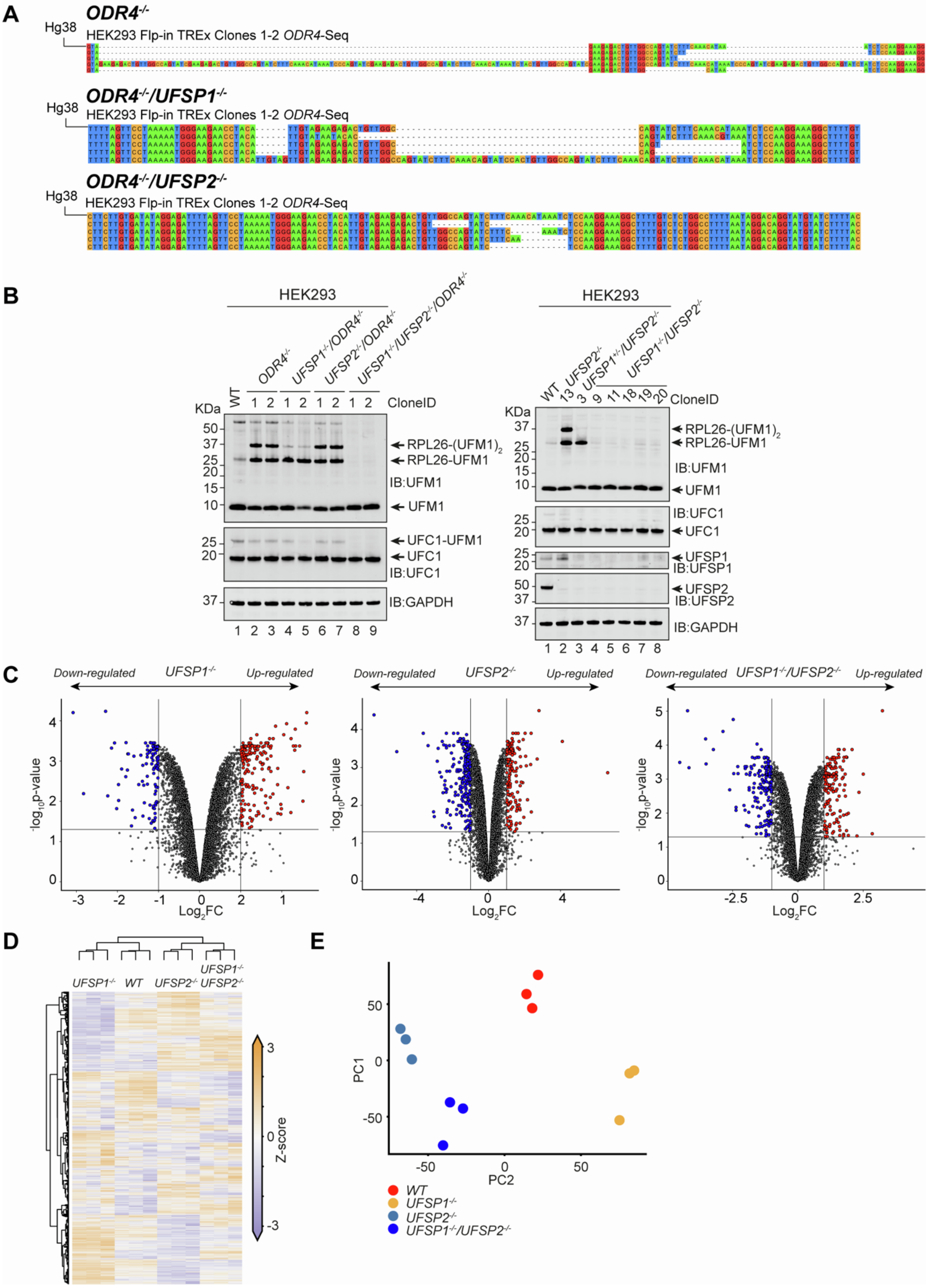

A
ODR4-/-
Hg38
HEK293 Flp-in TREx Clones 1-2 ODR4-Seq
ODR4-/-/UFSP1-/-
ODR4-/-/UFSP2-/-
B
HEK293
WT
ODR4-/-
UFSP1-/-/ODR4-/-
UFSP2-/-/ODR4-/-
UFSP1-/-/UFSP2-/-/ODR4-/-
CloneID
KDa
RPL26-(UFM1)2
RPL26-UFM1
IB:UFM1
UFM1
UFC1-UFM1
UFC1
IB:UFC1
IB:GAPDH
UFSP2-/-
UFSP1+/-/UFSP2-/-
UFSP1-/-/UFSP2-/-
UFSP1
IB:UFSP1
UFSP2
IB:UFSP2
C
Down-regulated
Up-regulated
UFSP1-/-
UFSP2-/-
UFSP1-/-/UFSP2-/-
-log10p-value
Log2FC
D
UFSP1-/-
WT
UFSP2-/-
UFSP1-/- UFSP2-/-
Z-score
E
PC1
PC2
WT
UFSP1-/-
UFSP2-/-
UFSP1-/-/UFSP2-/-

Figure-S6. ODR4 function in UFMylation. Related to Figure 6. (A) Representative sequencing analysis of the *ODR4* locus. Shown are two alleles identified from 8 sequencing reactions aligned to the Hg38 reference genome. Clone-ID (MRC-PPU internal) for analysis in Fig-6A is as follows; *UFSP1$^{-/-}$* (Clone-1); *UFSP2$^{-/-}$* (Clone-13; as in Fig-S1); *UFSP1$^{-/-}$/UFSP2$^{-/-}$* (Clone-11); *ODR4$^{-/-}$* (Clone-1); *ODR4$^{-/-}$/UFSP1$^{-/-}$* (Clone-1); *ODR4$^{-/-}$/UFSP2$^{-/-}$* (Clone-1). Full sequencing data is available on request from the study lead author. **(B)** Immunoblot analysis of the indicated clones. Clone-3 (D) is heterozygous deficient for *UFSP1* and shows a selective defect in the second UFM1 modification on RPL26 (K134). **(C)** Data Independent Acuisition (DIA) quantitative proteomics of indicated knockout cell lines. Volcano plots showing differential expression analysis (LIMMA). **(D)** Heatmap analysis showing Z-score for proteins passing the statistical threshold relative to unmodified HEK293 cells (LIMMA/Benjamini & Hochberg adjusted p-value <0.05). **(E)** Principal component analysis of proteomics data (p_{adj}<0.05). Each dot of the same color is a technical replicate.

Figure-S7

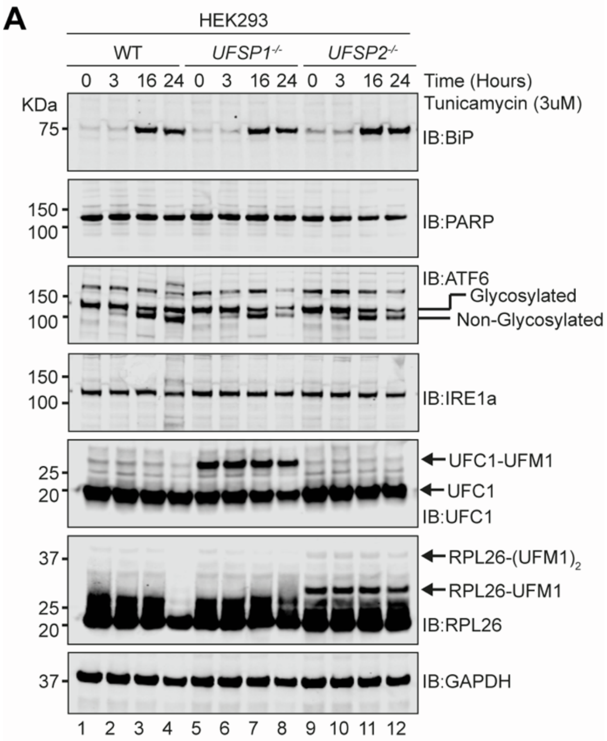

Figure-S7. Canonical ER-stress pathways. Related to Figure 7. (A) Time course immunoblot analysis of cell lysates from *UFSP1*$^{-/-}$ and *UFSP2*$^{-/-}$ cell lines stimulated with 30μM tunicamycin for the indicated time points.
